# Intracoronary versus intravenous glycoprotein IIb/IIIa inhibitors during primary percutaneous coronary intervention in patients with STEMI: a systematic review and meta-analysis

**DOI:** 10.1186/s12959-023-00519-x

**Published:** 2023-07-14

**Authors:** JongSung Hahn, Jinyoung Jeon, Min Jung Geum, Hyun Woo Lee, Jaekyu Shin, Woo-Young Chung, Yun Mi Yu, Young-Mi Ah

**Affiliations:** 1 College of Pharmacy, Jeonbuk National University, Jeonju, Republic of Korea; 2 Department of Pharmaceutical Medicine and Regulatory Sciences, Colleges of Medicine and Pharmacy, Yonsei University, Incheon, Republic of Korea; 3 Department of Pharmacy and Yonsei Institute of Pharmaceutical Sciences, College of Pharmacy, Yonsei University, Incheon, Republic of Korea; 4 Department of Pharmacy, National Cancer Center Hospital, Goyang, Republic of Korea; 5 Department of Pharmacy, Severance Hospital, Yonsei University Health System, Seoul, Republic of Korea; 6 Department of Clinical Pharmacy, School of Pharmacy, University of California San Francisco, San Francisco, CA USA; 7 Department of Internal Medicine, Seoul National University Boramae Medical Center and College of Medicine, Seoul National University, Seoul, Republic of Korea; 8 College of Pharmacy, Yeungnam University, 280 Daehak-Ro, Gyeongsan, Gyeongsangbuk-do 38541 Republic of Korea; 9 College of Pharmacy, Yonsei University, 85 Songdogwahak-ro, Yeonsu-gu, Incheon, 21983 Republic of Korea

**Keywords:** ST-elevation myocardial infarction, Percutaneous coronary intervention, Glycoprotein IIb/IIIa inhibitor, Intracoronary administration

## Abstract

**Background:**

Intracoronary (IC) administration of glycoprotein IIb/IIIa inhibitors (GPIs) has been studied as an adjunctive therapy to improve outcomes in patients with ST-segment elevation myocardial infarction (STEMI) undergoing percutaneous coronary intervention. In this systematic review and meta-analysis, we aimed to evaluate the efficacy and safety of IC administration of GPIs compared with those of intravenous (IV) administration in patients with STEMI.

**Methods:**

We searched the MEDLINE, Embase, and Cochrane CENTRAL databases for relevant studies published before September 21, 2022. In total, 22 randomized controlled trials involving 7,699 patients were included.

**Results:**

The proportions of patients achieving thrombolysis in myocardial infarction grade 3 flow, myocardial blush grade 2/3, and complete ST-segment resolution were significantly higher in the IC group than in the IV group. Major adverse cardiac events (MACE) (RR: 0.54, 95% CI: 0.37–0.80) and heart failure (RR: 0.48, 95% CI: 0.25–0.91) within 1 month were significantly lower in the IC group than in the IV group; however, after 6 months, no difference was observed in MACE risk. Additionally, the risks of death and bleeding did not differ between the two routes of administration.

**Conclusions:**

When considering adjunctive GPI administration for patients with STEMI, the IC route may offer greater benefits than the IV route in terms of myocardial reperfusion and reduced occurrence of MACE and heart failure within 1 month. Nonetheless, when making decisions for IC administration of GPIs, the absence of a benefit for bleeding risk and difficulty accessing the administration route should be considered.

**Supplementary Information:**

The online version contains supplementary material available at 10.1186/s12959-023-00519-x.

## Background

ST-segment elevation myocardial infarction (STEMI) is associated with a greater risk of cardiac death and serious complications, such as cardiac failure and arrhythmias, than other acute coronary syndromes. Therefore, myocardial reperfusion must be achieved before irreversible consequences occur. Primary percutaneous coronary intervention (PPCI) is the cornerstone therapy for STEMI [1]; however, myocardial reperfusion after PPCI can often be inadequate owing to the no-reflow phenomenon [2], which is a risk factor for cardiac death and other adverse cardiac events [3]. Specifically, an inflammatory response, oxygen free radicals, embolization, and platelet aggregation have been proposed as mechanisms of the no-reflow phenomenon. Prevention strategies for the phenomenon include direct stenting, thrombectomy, and glycoprotein IIb/IIIa inhibitor (GPI) administration.

Current guidelines recommend using GPIs in patients with a high thrombus burden to minimize the risk of the no-reflow phenomenon [4, 5]. The available GPIs include abciximab, a large monoclonal antibody that binds to glycoprotein IIb/IIIa receptor, and tirofiban and eptifibatide, non-peptide small molecules specific for glycoprotein IIb/IIIa receptor. GPIs are indicated to prevent cardiac ischemic complications in patients with non-ST-elevation acute coronary syndrome undergoing PCI and used off-label in patients with STEMI undergoing PCI. The contraindications for GPIs include hypersensitivity, active abnormal bleeding, and hemorrhagic stroke within 1 month. Among GPIs, abciximab is no longer readily available to clinicians in the United States and many European countries.

GPIs are potent antiplatelet agents that block the final common pathway of platelet aggregation and are generally administered via the intravenous (IV) route [1]. However, IV administration may result in low concentrations of GPI in vulnerable myocardial areas. Considering these limitations, several randomized controlled trials (RCTs) [6–8] have evaluated the efficacy of intracoronary (IC) administration of GPIs.

The Abciximab Intracoronary versus Intravenous Drug Application in STEMI (AIDA STEMI) trial, which included 2,067 patients, reported a non-significant difference in composite endpoints, including death, reinfarction, or congestive heart failure between IC and IV administration routes within 90 days of PPCI [6]. Similarly, a study on eptifibatide reported no difference in mortality risk, myocardial infarction recurrence, post-PCI reperfusion, and ST-segment resolution between the two routes of administration during a 1-month follow-up [7]. In contrast, a recent study by Ma et al. reported that, compared with IV administration, IC administration of tirofiban significantly reduced the risk of microvascular obstruction and left ventricular remodeling at 6 months but did not reduce mortality at 1 year [8].

Several systematic reviews and meta-analyses have compared IC and IV administration of GPIs. However, these reviews focused on only one type of GPI [9–11] or included studies that used abciximab [12–15]; therefore, the applicability of these studies in clinical practice might be limited. The most recent meta-analysis included studies published before April 11, 2017 [15]; since then, new RCTs comparing the efficacy and safety of GPIs administered via the IC and IV routes have been conducted [7, 8, 16]. Therefore, we aimed to evaluate and update the efficacy and safety of IC administration of GPIs compared with those of IV administration in patients with STEMI.

## Methods

This study followed the guidelines recommended by the Preferred Reporting Items for Systematic Reviews and Meta-analyses 2020 (PRISMA 2020) (Supplementary Table 1) [17]. The study protocol is available from the PROSPERO database (CRD42022375793). Two investigators (JH and JJ) independently performed the literature search, study selection, data extraction, and quality assessment. Discrepancies, if any, were resolved by two other investigators (YMY and YA).

### Search Strategy

The MEDLINE, Embase, and Cochrane CENTRAL electronic databases were systematically searched for relevant studies published before September 21, 2022, using a combination of medical subject headings and the keywords “STEMI,” “PCI,” “IC,” and “glycoprotein IIb/IIIa inhibitor.” The complete search strategy is shown in Supplementary Table 2.

### Study selection

Studies were considered eligible if they met the following inclusion criteria: (1) population: patients with STEMI undergoing PPCI; (2) intervention: adjunctive IC administration of GPIs; (3) comparison: adjunctive IV administration of GPIs; (4) outcomes: myocardial reperfusion and/or clinical outcomes; and (5) study design: RCTs. The following studies were excluded: (1) non-human studies, including animal and in vitro studies; (2) reviews, meta-analyses, and ongoing studies; (3) non-randomized studies or case reports; (4) studies available only in the form of abstracts or posters; and (5) publications in languages other than English.

### Data extraction

Eligible studies were reviewed, and the following data were extracted using a standardized extraction form: first author, publication year, country, study design, number of patients, sex, age, medical history, comorbidities (diabetes, dyslipidemia, and hypertension), smoking status, time from symptom onset to randomization, STEMI characteristics (preprocedural thrombolysis in myocardial infarction [TIMI] grade 0/1 flow, anterior infarction, and multivessel involvement), type of GPI (abciximab, tirofiban, and eptifibatide), dosing regimen, PCI procedures, follow-up duration, and study outcomes.

### Study outcomes

The primary study outcome was the incidence of myocardial reperfusion assessed using coronary reperfusion indices, including TIMI grade 3 flow, myocardial blush grade 2/3 (MBG 2/3), TIMI myocardial perfusion grade 3 (TMPG 3), corrected TIMI frame count (cTFC), and complete ST-segment resolution (STR) after PCI. The secondary study outcomes were clinical outcomes, including left ventricular ejection fraction (LVEF) improvement and incidence of major adverse cardiac events (MACE), heart failure, reinfarction, target vessel revascularization (TVR), stroke, cardiac or all-cause death, and bleeding events during the follow-up period. MACE was defined as a composite of acute myocardial infarction, TVR, and cardiovascular mortality. Bleeding events were classified as major or minor bleeding and defined according to either the TIMI bleeding classification or the Global Utilization of Streptokinase and Tissue Plasminogen Activator for Occluded Coronary Arteries criteria [18].

### Analyses

Pooled risk ratios (RRs) with 95% confidence intervals (CIs) and pooled standardized mean differences (SMDs) with 95% CIs were computed using the Mantel–Haenszel and generic inverse-variance methods, respectively [19]. Heterogeneity was assessed using the *I*^2^ statistic, with the desired threshold set at *I*^2^ > 50% [20]. A common-effect model was used in the absence of significant heterogeneity, and a random-effects model was employed when significant heterogeneity was present [21].

We conducted separate subgroup and meta-regression analyses and evaluated differences in the incidence of myocardial reperfusion and clinical outcomes between the IC and IV groups according to the time from symptom onset to randomization (≤ 6 h vs. > 6 h), the type of GPI, individual GPIs, the status of maintenance therapy with abciximab, the type of P2Y12 inhibitors, and based on the condition that > 80% of patients were undergoing thrombectomy. The meta-regression analysis was conducted according to the proportion of patients with current smoking status, comorbidities, and STEMI characteristics. Moreover, the sensitivity analysis was performed by removing low-quality studies and one study per analysis (leave-one-out) and adding each study in the order of sample size and year of publication to determine the robustness of the results.

The quality assessment of each included study was conducted using the Risk of Bias 2 (RoB 2) tool for RCTs [22]. Publication bias in each outcome was examined using funnel plots and Egger’s regression test when the number of eligible studies was six or more. Statistical significance was set at *P* < 0.05. The meta-module in R (version 4.2.1; R Foundation for Statistical Computing, Vienna, Austria) was used for statistical analyses.

## Results

### Study selection

Supplementary Fig. 1 shows the flow diagram of study selection according to the PRISMA 2020 guidelines. After excluding duplicates, 347 articles were screened for relevance based on their titles and abstracts, and 246 were excluded. The remaining 101 articles were assessed for eligibility through a full-text evaluation, and 22 RCTs with 7,699 patients were finally selected.

### Study characteristics

Table 1 summarizes the characteristics of the study protocols of the 22 RCTs. The number of participants ranged from 40 to 2,065 per study, and the follow-up period ranged from 1 month to 1 year. Only four studies (18%) administered the treatment within 6 h after symptom onset. Furthermore, the proportion of patients who underwent thrombectomy during PCI ranged from 0 to 100%, as reported in 14 studies. The GPIs included abciximab (13 studies) [6, 23–34] and small molecules (7 studies), such as eptifibatide (3 studies) [7, 35, 36] and tirofiban (4 studies) [8, 16, 37, 38]. Moreover, two studies compared abciximab and eptifibatide [39, 40].

**Table 1:** Characteristics of included studies

First author, year (country)	Sample size (IC/IV group)	Follow-up duration, months	Symptom onset^a^, hours	Thrombectomy	Stenting (DES)	P2Y12 inhibitors	GP-IIb/IIIa inhibitors	IC bolus dose^b^	Maintenance IV infusion
Bellandi, 2004 (Italy) [23]	22/23	1	≤ 6	NA	100% (NA)	TP	Abciximab	Standard	Y
Thiele, 2008 (Germany) [24]	77/77	1	≤ 12	NA	Almost all (NA)	C	Abciximab	Standard	Y
Dominguez-Rodriguez, 2009 (Spain) [25]	25/25	1	≤ 6	100%	100% (NA)	C	Abciximab	Standard	Y
Bertrand, 2010 (Canada) [26]	53/52	12	≤ 6	42%	NA (NA)	C	Abciximab	Standard	Y
Gu, 2010 (Netherlands) [27]	271/263	1	≤ 12	98%	95% (NA)	C or P	Abciximab	Standard	N
Eitel, 2011 (Germany) [28]	77/77	6	≤ 12	NA	NA (NA)	C	Abciximab	Standard	Y
Iversen, 2011 [30 days] (Denmark) [29]	185/170	1	≤ 12	0%	95% (80%)	C	Abciximab	Standard	Y
Iversen, 2011 [1 year] (Denmark) [30]	185/170	12	≤ 12	0%	95% (80%)	C	Abciximab	Standard	Y
Kirma, 2012 (Turkey) [37]	25/24	6	≤ 12	NA	100% (NA)	C	Tirofiban	Standard	Y (IV group only)
Thiele, 2012 (Germany) [6]	1032/1033	3	≤ 12	20%	Almost all (NA)	C or P	Abciximab	Standard	Y
Desch, 2013 (Germany) [31]	925/921	12	≤ 12	20%	Almost all (NA)	C or P	Abciximab	Standard	Y
Eitel, 2013 (Germany) [32]	394/401	12	≤ 12	24%	98% (42%)	C or P	Abciximab	Standard	Y
Namazi, 2013 (Iran) [39]	20/20	Until discharge	≤ 12	68%	100% (65%)	C	Abciximab (IC group) Eptifibatide (IV group)	Standard	N
Pellicori, 2013 (Italy) [40]	38/39	12	≤ 12	0%	100% (NA)	C	Abciximab or eptifibatide	Abciximab: Standard Eptifibatide: Low	Y
Secco, 2014 (Italy) [33]	47/42	6	≤ 12	25%	NA	C	Abciximab	Standard	N
Esfandi, 2016 (Iran) [35]	36/38	Until discharge	NA	NA	100% (100%)	C	Eptifibatide	Standard	Y
Sanati, 2017 (Iran) [7]	32/32	Until discharge	≤ 12	NA	NA (10%)	C	Eptifibatide	Standard	Y
Bedjaoui, 2019 (Algeria) [34]	78/82	6	≤ 12	82%	96% (NA)	C	Abciximab	Standard	Y
Nab, 2019 (Egypt) [36]	50/50	1	≤ 12	92%	100% (0%)	C	Eptifibatide	Standard	Y
Ma, 2020 (China) [8]	106/102	12	≤ 12	NA	100% (NA)	C	Tirofiban	Low	Y
Tang, 2022 [DM] (China) [38]	100/100	15 days	≤ 2	NA	NA	NA	Tirofiban	Low	Y
Tang, 2022 (China) [16]	90/90	6	≤ 12	26%	Almost all (NA)	TG	Tirofiban	Low	Y

^a^ Time from symptom onset to randomization

^**b**^ Standard dose for IC bolus: abciximab 0.25 mg/kg, eptifibatide 180 mcg/kg double-bolus (10-min interval), and tirofiban 25 mcg/kg

Abbreviations: C, clopidogrel; DES, drug-eluting stent; GP, glycoprotein; IC, intracoronary; IV, intravenous; N, no in both groups; NA, not available; P, prasugrel; TG, ticagrelor; TP, ticlopidine; Y, yes in both groups

Patient baseline characteristics are presented in Supplementary Table 3. The mean age of the participants ranged from 51.0 to 68.0 years, with males comprising over two-thirds of the participants in all the studies. The proportion of current smokers ranged from 31.0 to 73.5%, while that of patients with diabetes varied from 9.0 to 100%; notably, Tang et al. included only patients with diabetes [38]. Finally, the proportion of patients with anterior infarction and multivessel involvement (≥ 2 vessels) ranged from 39.4 to 100.0% and 33.2–70.0%, respectively.

### Myocardial reperfusion

**Fig. 1:**
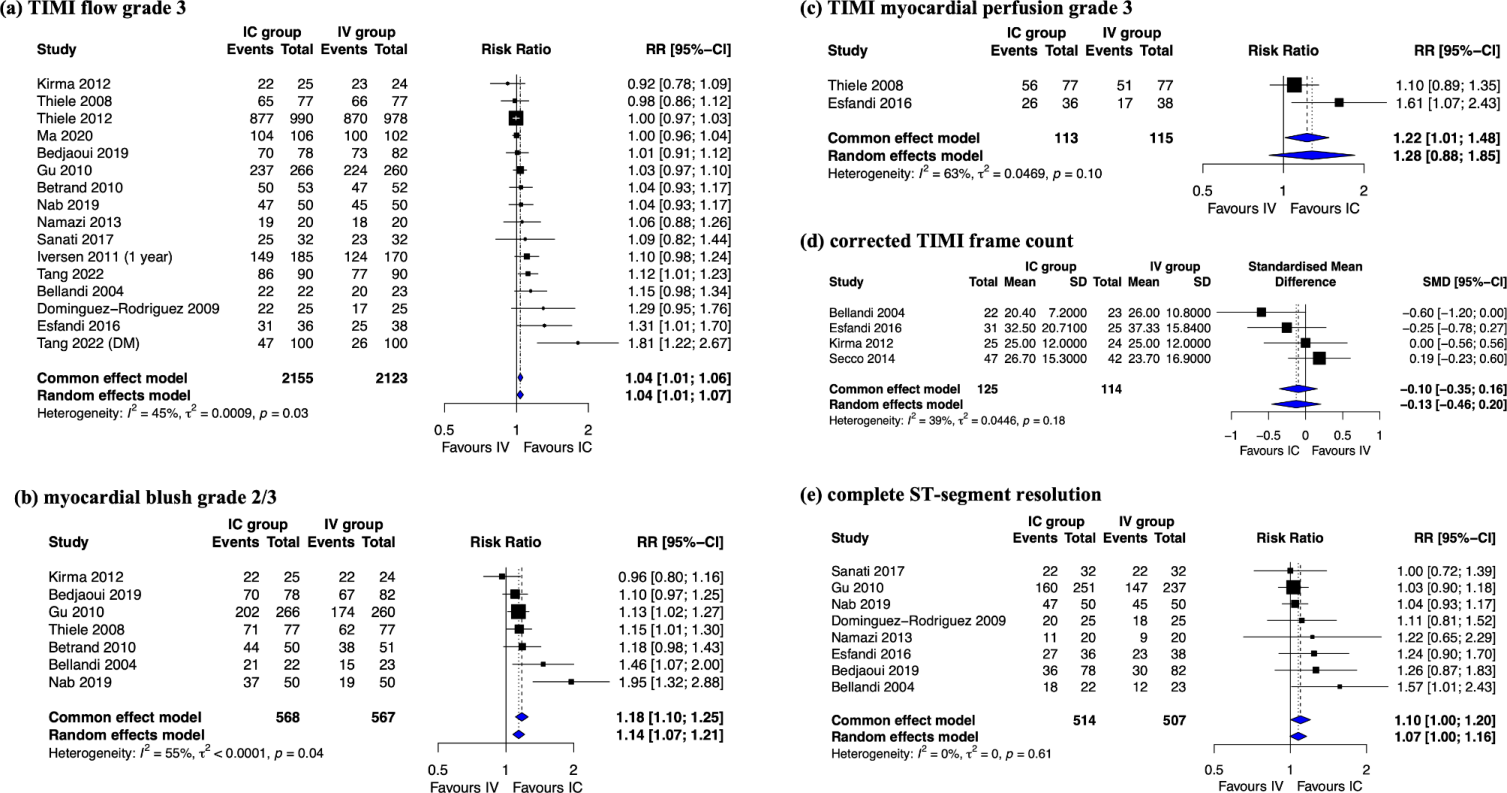
Forest plot of the incidence of myocardial reperfusion: intracoronary vs. intravenous glycoprotein IIb/IIIa inhibitors (a) Thrombolysis in myocardial infarction (TIMI) grade 3 flow, (b) myocardial blush grade 2/3, (c) TIMI myocardial perfusion grade 3 (d) corrected TIMI frame count, and (e) complete ST-segment resolution

TIMI grade 3 flow [6–8, 16, 23–27, 30, 34–39], MBG 2/3 [23, 24, 26, 27, 34, 36, 37], TMPG 3 [24, 35], cTFC [23, 33, 35, 37], and complete STR [7, 23, 25, 27, 34–36, 39] were reported in 16, 7, 2, 4, and 8 studies, respectively. The proportions of patients achieving TIMI grade 3 flow, MBG 2/3, and complete STR were significantly higher in the IC group than in the IV group (RR: 1.04, 95% CI: 1.01–1.06; RR: 1.14, 95% CI: 1.07–1.21; RR: 1.10, 95% CI: 1.00–1.20, respectively). Although not statistically significant, the proportion of patients achieving TMPG 3 and cTFC also showed a favorable trend in the IC group compared with that in the IV group (Fig. 1).

**Fig. 2:**
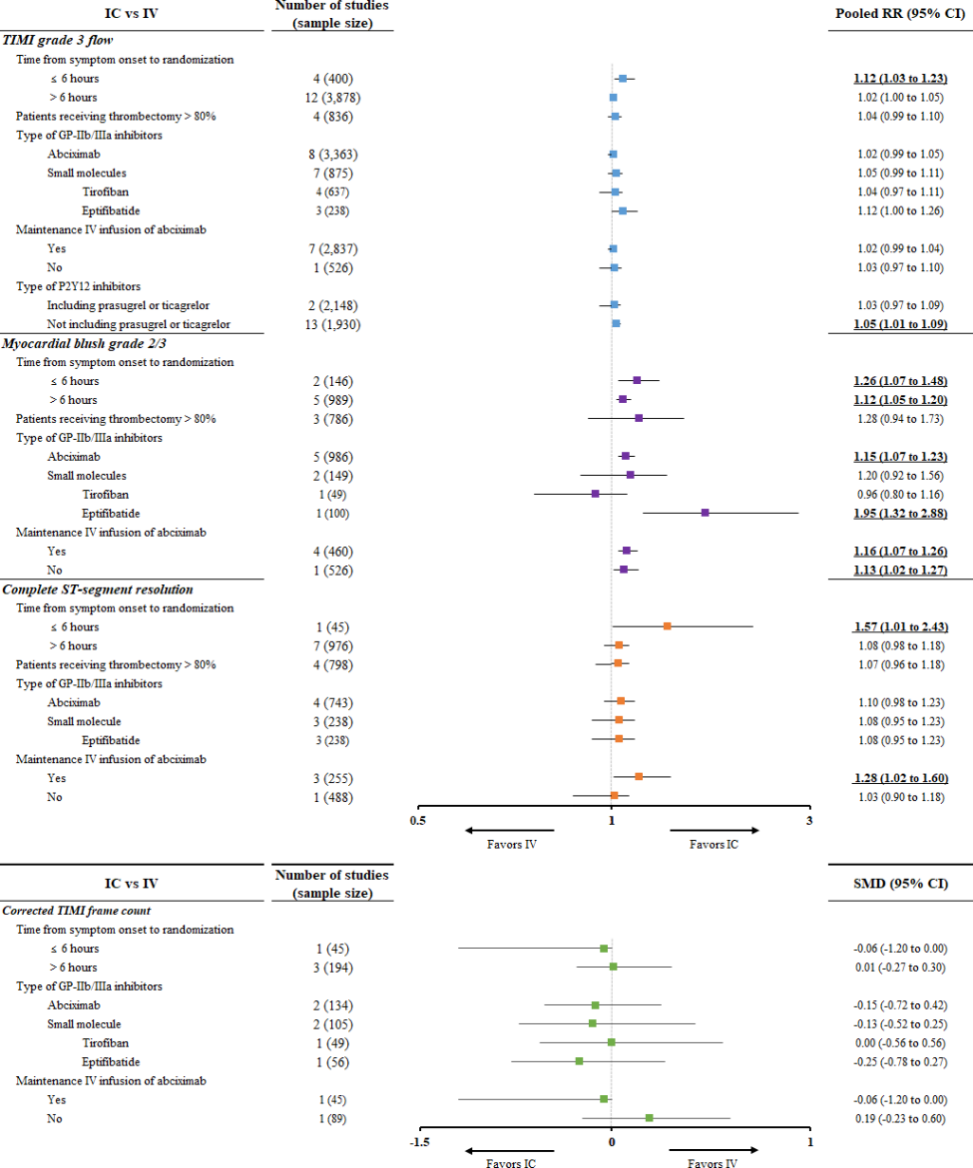
Subgroup analysis of the incidence of myocardial reperfusion

IC administration was superior to IV administration in both subgroups according to the symptom onset to randomization time (≤ 6 h and > 6 h) for TIMI grade 3 flow and MBG (Fig. 2). The meta-regression analysis showed that the incidence of myocardial reperfusion did not significantly differ according to the patients’ baseline characteristics of current smoking, comorbidities, and specific STEMI characteristics. When the proportion of patients with diabetes and dyslipidemia was higher, achievements of TIMI grade 3 flow (beta coefficient: 0.0034, 95% CI: 0.0004–0.0064) and MBG 2/3 (beta coefficient: 0.0054, 95% CI: 0.0000–0.0107) were significantly higher with IC administration than with IV administration, respectively. However, these results have limited clinical implications because the beta coefficient is very low (Supplementary Table 4).

### Clinical outcomes

**Fig. 3:**
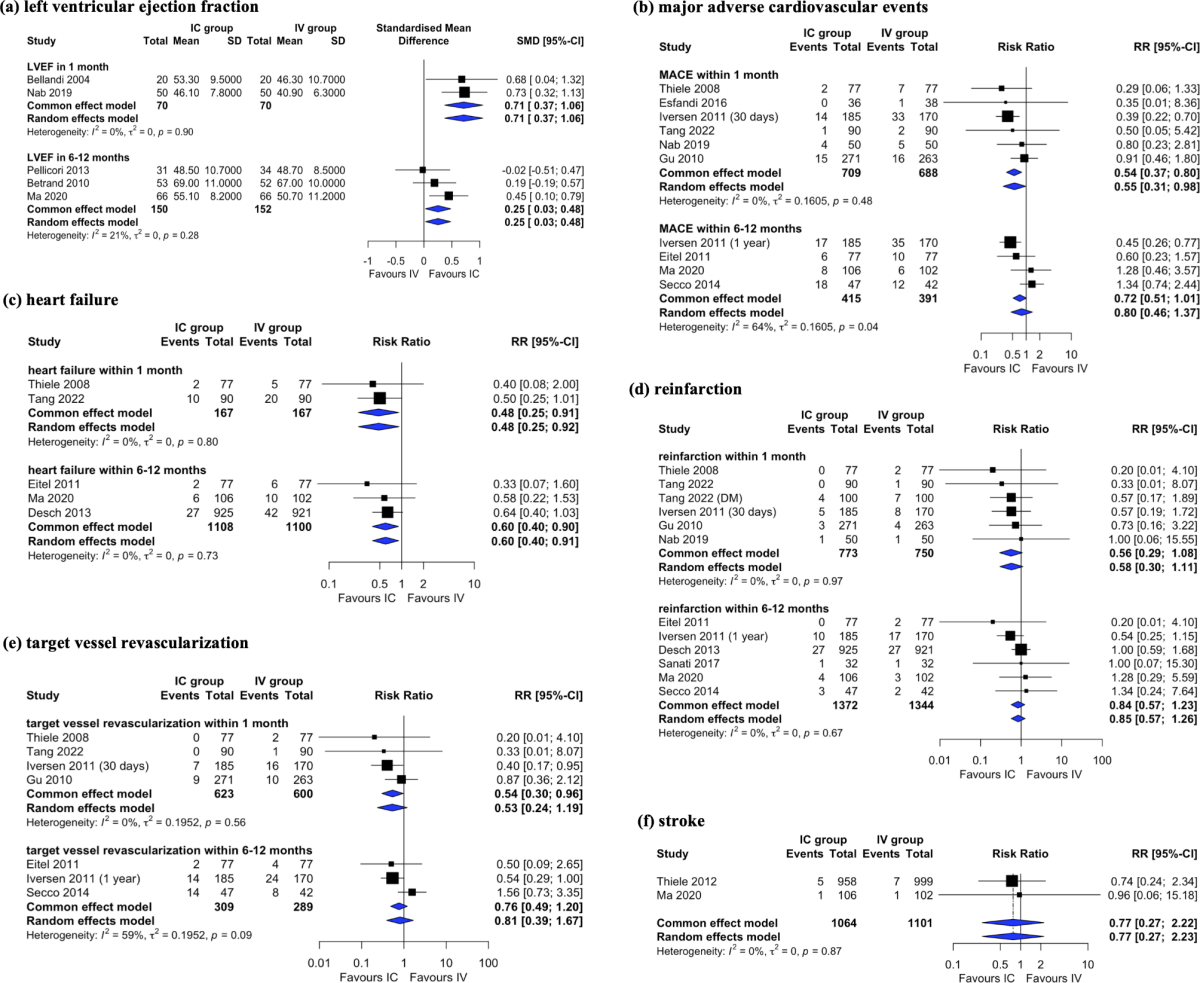
Forest plot comparing LVEF improvement and MACE risks: intracoronary vs. intravenous glycoprotein IIb/IIIa inhibitors (a) LVEF, (b) MACE, (c) heart failure, (d) reinfarction, (e) target vessel revascularization, and (f) stroke

Figure 3 shows a comparison of clinical outcomes between IC and IV administration of GPIs. LVEF improvement during the follow-up period was compared between five studies [8, 23, 26, 36, 40]. The SMD of LVEF after 1 month was higher in the IC group than in the IV group (SMD: 0.71, 95% CI: 0.37–1.06). The difference in the LVEF after 6 months or longer was also higher in the IC group, with borderline significance (SMD: 0.25, 95% CI: 0.03–0.48). The risks of MACE [8, 16, 24, 27–31, 33, 35, 36, 38], heart failure [8, 16, 24, 28, 31], reinfarction [7, 8, 16, 24, 27–31, 33, 36, 38], TVR [16, 24, 27–30, 33], and stroke [6, 8] were reported in 10, 5, 12, 7, and 2 studies, respectively. The incidence of MACE within 1 month was significantly lower in the IC group than in the IV group (RR: 0.54, 95% CI: 0.37–0.80). However, the incidence of MACE within 6–12 months did not differ between the two groups. Furthermore, the risk of heart failure was significantly lower in the IC group than in the IV group, regardless of the follow-up duration (RR: 0.48, 95% CI: 0.25–0.91 within 1 month; RR: 0.60, 95% CI: 0.40–0.90 within 6–12 months). The risks of reinfarction, TVR, and stroke did not differ between the IC and IV groups.

The risks of mortality [6–8, 16, 27, 28, 30, 31, 33, 35, 36, 38] and bleeding events [6, 8, 16, 24, 26, 27, 29, 33–36, 39] were reported in 12 studies each. There were no significant differences in the risks of all-cause death and cardiac death between the two groups (RR: 0.88, 95% CI: 0.64–1.21; RR: 1.07, 95% CI: 0.73–1.57) (Supplementary Fig. 2). Similarly, no significant difference between the two groups was observed in the risks of major and minor bleeding events (RR: 1.10, 95% CI: 0.73–1.64; RR: 0.82, 95% CI: 0.62–1.06) (Supplementary Fig. 3).

**Fig. 4:**
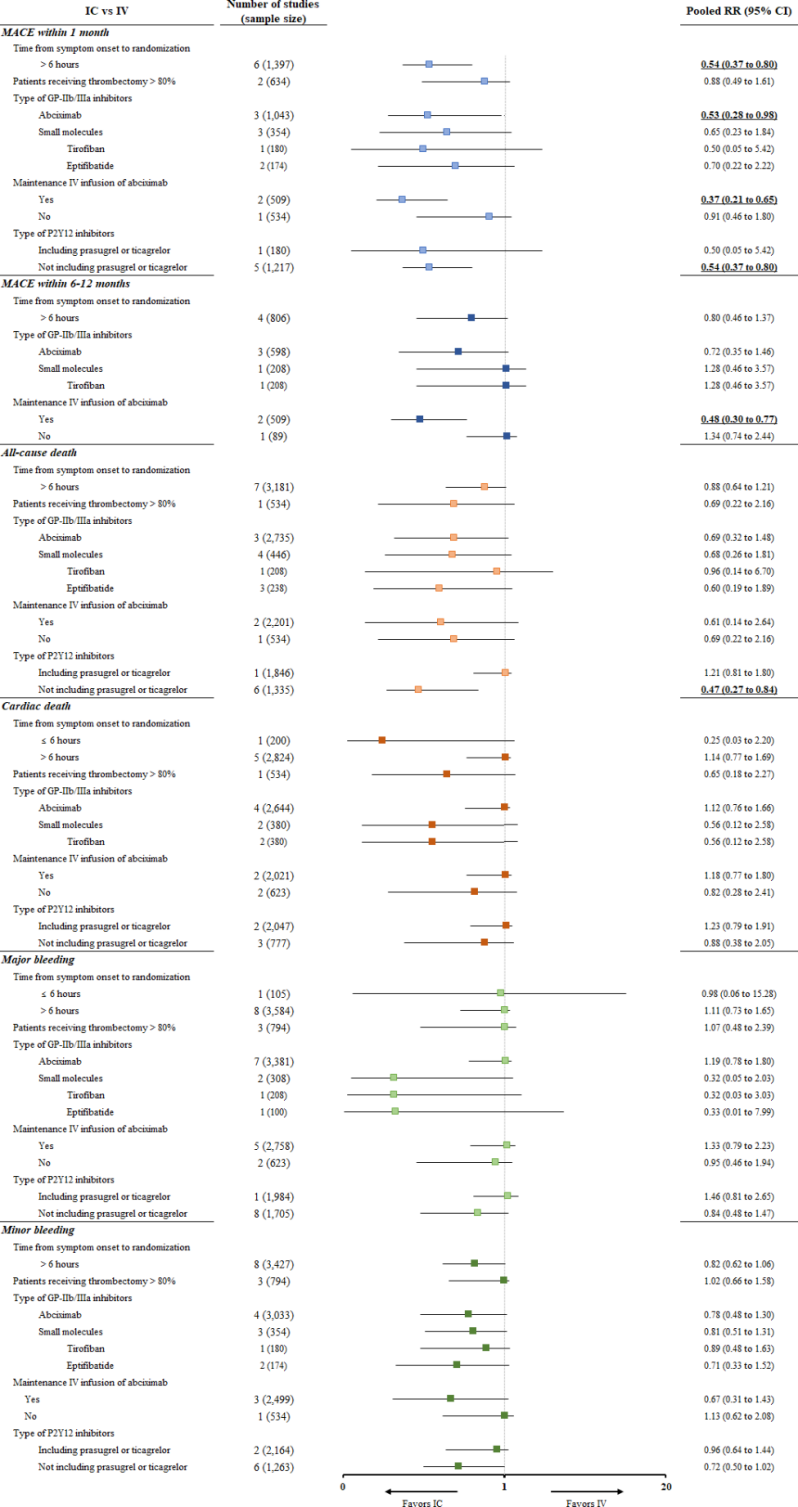
Subgroup analysis of clinical outcomes risks

MACE occurrence within 1 month was evaluated only in patients with the symptom onset to randomization time > 6 h; IC administration had a lower risk of MACE than did IV administration in the subgroups receiving IC abciximab and maintenance IV infusion (Fig. 4). Furthermore, the meta-regression analysis showed that clinical outcomes did not significantly differ according to the patients’ baseline characteristics of current smoking, comorbidities, and specific STEMI characteristics (Supplementary Table 4).

### ***Risk of Bias Assessments***

The risk of bias assessment revealed that 10 of the 22 RCTs (45.5%) were of some concern (Supplementary Fig. 4). The reason for the downgrading was that the method of randomization and/or allocation concealment was not described (Supplementary Table 5). Visual inspection of the funnel plot and Egger’s test revealed a publication bias in the outcomes of TIMI grade 3 flow and complete STR but not in the other six outcomes (Supplementary Fig. 5).

### Sensitivity analysis

The sensitivity analysis results based on the quality assessment are presented in Supplementary Table 6. When analyzing the studies without the concern of bias, the results were similar to the overall findings. The sensitivity analysis showed no effect of leave-one-out, sample size, or year of publication on the findings (Supplementary Figs. 6–8). For outcomes pooled from a small number of studies, such as those of TMPG 3 and LVEF, robustness could not be fully assessed.

## Discussion

Our systematic review and meta-analysis compared the efficacy and safety of IC and IV administration of a GPI bolus in patients with STEMI undergoing PPCI. Compared with IV administration, IC administration significantly increased TIMI grade 3 flow (RR: 1.04; 95% CI: 1.01–1.06), MBG 2/3 (RR: 1.14; 95% CI: 1.07–1.21), and complete STR (RR: 1.10; 95% CI: 1.00–1.20). IC administration was more effective in improving the LVEF and reducing the incidence of heart failure, regardless of follow-up time; however, it was more effective than IV administration in reducing MACE incidence only within a follow-up time of 1 month. No between-group significant differences were observed in the occurrence of all-cause death, cardiac death, or bleeding events.

IC administration of GPIs may result in a high local concentration, leading to increased platelet GP IIb/IIIa receptor occupancy levels and inhibition of platelet aggregation in the epicardial arteries and microvasculature [41, 42]. Meta-analyses conducted before 2017 [10–12] mainly included one type of GPI: abciximab. Kubica et al. and De Luca G et al. reported that IC abciximab had no benefits in terms of reducing mortality, while De Luca G et al. and Wang et al. found a significant improvement in myocardial perfusion. Furthermore, Wang et al. reported a reduction of 30-day MACE. In the most recent meta-analysis of 14 RCTs, Elbadawi found a significantly higher achievement of TIMI grade 3 flow, MBG 2/3, complete STR, improvement of LVEF, and reduction of short-term (≤ 3 months) MACE with IC administration of GPIs than with IV administration [15]. In our meta-analysis, we broadened the scope to more than one type of GPI, and we assessed MACEs within 1 month and within 6–12 months. Improvement in myocardial perfusion with IC route was consistent with the findings of previous meta-analyses; the significant improvement in both ischemic time subgroups was a new finding. MACE and HF within 1 month were significantly lower with IC route; however, we did not find benefits within 6–12 months.

Our findings revealed that, compared with IV administration, IC administration of GPIs significantly increased the chance of complete perfusion (TIMI grade 3 flow and MBG 2/3, indicative of macrovascular and microvascular reperfusion, respectively). This finding can mainly be explained by the high local platelet inhibitor concentration caused by the IC injection. Similarly, the incidence of restored myocardial reperfusion, defined as complete STR, increased with IC administration. Nonetheless, no difference was observed between TMPG 3 and cTFC, possibly because of the small number of included studies (two and four, respectively).

In our meta-analysis, compared with IV administration, IC administration improved the LVEF and reduced the incidence of heart failure, regardless of follow-up duration. A high rate of complete perfusion is known to decrease the size of the infarcted parts of the myocardium and subsequently increase heart contractility and LVEF [43]. Furthermore, decreased heart failure incidence, an important target of GPI therapy, might be related to LVEF improvement. This result is consistent with the main findings of Tang et al. [16] and AIDA STEMI [6]. However, caution should be exercised when interpreting the improvement in LVEF, as studies reporting LVEF did not present a baseline LVEF or measure the degree of change in LVEF. Improved TIMI flow or myocardial reperfusion is known to be closely related to MACE reduction in PCI patients [44, 45]. Nevertheless, in our results, IC administration significantly reduced MACE incidence only within 1 month. This means that the low incidence rate of MACE with IC administration is not sustained in the long-term.

All GPIs increased the risk of bleeding owing to antiplatelet activity. We found no significant difference in the number of bleeding events between the IC and IV groups, indicating that IC administration has no advantage in terms of reducing bleeding risk. This might be explained by the fact that the two groups received the same drug at the same total dosage and duration in each study. Similarly, there was no difference between IC and IV groups in terms of mortality risk.

Our subgroup analysis indicated that the IC route was superior to the IV route in both the ischemic time subgroups of ≤ 6 h and > 6 h for TIMI grade 3 flow and MBG 2/3. This new finding suggests that IC GPIs could favorably affect myocardial reperfusion regardless of the ischemic time. We also divided the GPIs into two subgroups (abciximab and small molecules) for analyses, and no differences were found. However, MACE within 1 month was significantly reduced with abciximab but not with the small molecules. This might be associated with the large number of participants included in the studies of abciximab. Small-molecule GPIs are known to possess advantages such as improved platelet-fibrin thrombus penetration, enhanced platelet aggregation inhibition at the end of infusion, and greater cost-effectiveness [46, 47]. Hence, it is imperative to conduct large-scale trials evaluating small-molecule GPIs extensively. Once sufficient evidence is gathered, small-molecule GPIs could be considered a viable alternative in situations where abciximab is scarce.

Furthermore, sensitivity analysis showed that the significant outcomes following IC administration were not dependent on the results of individual studies. When AIDA, the largest trial to date, was removed, the overall result remained the same. The sensitivity analysis with the sample size, publication year, and RoB 2 also demonstrated the robustness of the results. Taken together, the results of this meta-analysis suggest that the use of IC over IV GPIs might be justified, although observed improvements were primarily related to myocardial reperfusion, and clinical results showed only marginal or short-term (within 1 month) improvements. However, the benefits of IC over IV administration should be investigated further through large-scale, high-quality RCTs, considering that the risks of bleeding and death were not different between the two groups and that the IC catheterization method is more complicated in a situation where early administration is essential for successful treatment.

This study had some limitations. First, our study results should be interpreted cautiously, given the wide CI and limited clinical outcome data. Second, we had no access to patient-level data, such as medical history (hypertension, dyslipidemia, diabetes mellitus, previous myocardial infarction, and current smoking status), or the ability to examine which patients benefited the most from IC administration. Study-level meta-regression analyses were performed; however, they revealed few clinical implications because of the low beta coefficient. Third, the concurrent use of P2Y12 inhibitors differed between studies. Specifically, the efficacy and safety of GPI injections might change with the development of more potent P2Y12 inhibitors, such as ticagrelor and prasugrel. Nonetheless, most of the studies included in our meta-analysis used clopidogrel, and we could not find a significant difference in the subgroup analysis according to the type of P2Y12 inhibitors. Further studies assessing the influence of potent P2Y12 inhibitors on the clinical outcomes of GPIs administered via IC and IV routes are required. Fourth, the subgroup analysis based on maintenance therapy after bolus administration showed few clinical implications because of the few included studies. Finally, many of the included studies raised concerns regarding the risk of bias. Nevertheless, the sensitivity analysis showed no discrepancy in the results according to the risk level. Fifth, although we performed subgroup analysis according to GPIs, no significant differences were identified among GPIs owing to the disparity in outcome reports and the small number of studies for each outcome. Therefore, further studies assessing the efficacy and safety of different IC GPIs are required.

## Conclusions

In conclusion, when considering adjunctive GPI administration for patients with STEMI, the IC route may offer greater benefits than the IV route in terms of post-PPCI myocardial reperfusion and reduced incidence of MACE and heart failure within 1 month. However, when making decisions for IC administration of GPIs, the absence of a benefit for bleeding risk and difficulty accessing the administration route should be considered.

## Electronic supplementary material

Below is the link to the electronic supplementary material.

Supplementary Material 1

## Data Availability

All data generated or analyzed during this study are included in this published article and its supplementary information files.

## List of abbreviations

AIDA STEMI: Abciximab Intracoronary versus Intravenous Drug Application in STEMI
CIs: Confidence intervals
cTFC: Corrected TIMI frame count
GPI: Glycoprotein IIb/IIIa inhibitors
IC: Intracoronary
IV: Intravenous
LVEF: Left ventricular ejection fraction
MACE: Major adverse cardiac events
MBG: Myocardial blush grade
PPCI: Primary percutaneous coronary intervention
PRISMA 2020: Preferred Reporting Items for Systematic Reviews and Meta-analyses 2020
RCT: Randomized controlled trials
RoB 2: Risk of Bias 2
SMD: Standardized mean differences
RRs: Pooled risk ratios
STEMI: ST-segment elevation myocardial infarction
STR: ST-segment resolution
TIMI: Thrombolysis in myocardial infarction
TMPG: TIMI myocardial perfusion grade
TVR: Target vessel revascularization
