# Supplementary material for: Intracoronary versus intravenous glycoprotein IIb/IIIa inhibitors during primary percutaneous coronary intervention in patients with STEMI: a systematic review and meta-analysis
Source: Thromb J. 2023 Jul 14;21:76. doi: 10.1186/s12959-023-00519-x (PMC10347711; doi:10.1186/s12959-023-00519-x)
Supplement: Supplementary file 1 — Supplementary Material 1 [file 12959_2023_519_MOESM1_ESM.docx]

**Intracoronary versus Intravenous glycoprotein-IIb/IIIa inhibitors during primary percutaneous coronary intervention in patients with STEMI: A systematic review and meta-analysis**

JongSung Hahn,^1†^ Jinyoung Jeon,^2,3,4†^ Min Jung Geum,^5^ Hyun Woo Lee, ^2,3^ Jaekyu Shin,^6^ Woo-Young Chung,^7^ Yun Mi Yu ^2,3*^ Young-Mi Ah^8*^

^1^College of Pharmacy, Jeonbuk National University, Jeonju, Republic of Korea

^2^Department of Pharmaceutical Medicine and Regulatory Sciences, Colleges of Medicine and Pharmacy, Yonsei University, Incheon, Republic of Korea

^3^Department of Pharmacy and Yonsei Institute of Pharmaceutical Sciences, College of Pharmacy, Yonsei University, Incheon, Republic of Korea

^4^Department of Pharmacy, National Cancer Center Hospital, Goyang, Republic of Korea

^5^Department of Pharmacy, Severance Hospital, Yonsei University Health System, Seoul, Republic of Korea

^6^Department of Clinical Pharmacy, School of Pharmacy, University of California San Francisco, San Francisco, CA, USA

^7^Department of Internal Medicine, Seoul National University Boramae Medical Center and College of Medicine, Seoul National University, Seoul, Republic of Korea

^8^College of Pharmacy, Yeungnam University, Gyeongsan, Republic of Korea

**Supplementary Materials**

Supplementary Table 1 Checklist for Preferred Reporting Items for Systematic Reviews and Meta-Analyses

Supplementary Table 2 Search strategy

Supplementary Table 3 Baseline patient characteristics

Supplementary Table 4 Meta-regression according to baseline patient characteristics

Supplementary Table 5 Detailed information regarding the risk of bias assessment according to the Risk of Bias 2 criteria

Supplementary Table 6 Sensitivity analysis based on quality assessment

Supplementary Figure 1 Flowchart of the study selection process

Supplementary Figure 2 Forest plot of mortality risk in intracoronary administration of glycoprotein IIb/IIIa inhibitors compared with that in intravenous administration. (a) all-cause death and (b) cardiac death

Supplementary Figure 3 Forest plot of bleeding risk after intracoronary administration of glycoprotein IIb/IIIa inhibitors compared with that after intravenous administration. (a) major bleeding and (b) minor bleeding

Supplementary Figure 4 Quality assessment of the risk of bias according to the Risk of Bias 2 criteria

Supplementary Figure 5 Funnel plot and Egger’s test for the incidence of myocardial reperfusion and clinical outcomes

Supplementary Figure 6 Sensitivity analysis with the leave-one-out method

Supplementary Figure 7 Sensitivity analysis with a small sample size

Supplementary Figure 8 Sensitivity analysis by publication year

Supplementary Table 1 Checklist for Preferred Reporting Items for Systematic Reviews and Meta-Analyses

| **Section/topic** | **#** | **Checklist item** | **Reported on page #** |
| --- | --- | --- | --- |
| **TITLE** |  |  |  |
| Title | 1 | Identify the report as a systematic review, meta-analysis, or both. | 1 |
| **ABSTRACT** |  | |  |
| Structured summary | 2 | Provide a structured summary including, as applicable: background; objectives; data sources; study eligibility criteria, participants, and interventions; study appraisal and synthesis methods; results; limitations; conclusions and implications of key findings; systematic review registration number. | 3 |
| **INTRODUCTION** |  |  |  |
| Rationale | 3 | Describe the rationale for the review in the context of what is already known. | 4 |
| Objectives | 4 | Provide an explicit statement of questions being addressed with reference to participants, interventions, comparisons, outcomes, and study design (PICOS). | 5 |
| **METHODS** | | | |
| Protocol and registration | 5 | Indicate if a review protocol exists, if and where it can be accessed (e.g., Web address), and, if available, provide registration information including registration number. | 5 |
| Eligibility criteria | 6 | Specify study characteristics (e.g., PICOS, length of follow-up) and report characteristics (e.g., years considered, language, publication status) used as criteria for eligibility, giving rationale. | 5-6 |
| Information sources | 7 | Describe all information sources (e.g., databases with dates of coverage, contact with study authors to identify additional studies) in the search and date last searched. | 5 |
| Search | 8 | Present full electronic search strategy for at least one database, including any limits used, such that it could be repeated. | Supplementary table 2 |
| Study selection | 9 | State the process for selecting studies (i.e., screening, eligibility, included in systematic review, and, if applicable, included in the meta-analysis). | 5-6 |
| Data collection process | 10 | Describe method of data extraction from reports (e.g., piloted forms, independently, in duplicate) and any processes for obtaining and confirming data from investigators. | 6 |
| Data items | 11 | List and define all variables for which data were sought (e.g., PICOS, funding sources) and any assumptions and simplifications made. | 6 |
| Risk of bias in individual studies | 12 | Describe methods used for assessing risk of bias of individual studies (including specification of whether this was done at the study or outcome level), and how this information is to be used in any data synthesis. | 7 |
| Summary measures | 13 | State the principal summary measures (e.g., risk ratio, difference in means). | 6 |
| Synthesis of results | 14 | Describe the methods of handling data and combining results of studies, if done, including measures of consistency (e.g., I^2^) for each meta-analysis. | 6-7 |
| Risk of bias across studies | 15 | Specify any assessment of risk of bias that may affect the cumulative evidence (e.g., publication bias, selective reporting within studies). | 7 |
| Additional analyses | 16 | Describe methods of additional analyses (e.g., sensitivity or subgroup analyses, meta-regression), if done, indicating which were pre-specified. | 7 |
| **RESULTS** | | | |
| Study selection | 17 | Give numbers of studies screened, assessed for eligibility, and included in the review, with reasons for exclusions at each stage, ideally with a flow diagram. | 7 |
| Study characteristics | 18 | For each study, present characteristics for which data were extracted (e.g., study size, PICOS, follow-up period) and provide the citations. | 8 |
| Risk of bias within studies | 19 | Present data on risk of bias of each study and, if available, any outcome level assessment (see item 12). | 9-10 |
| Results of individual studies | 20 | For all outcomes considered (benefits or harms), present, for each study: (a) simple summary data for each intervention group (b) effect estimates and confidence intervals, ideally with a forest plot. | 7-9 |
| Synthesis of results | 21 | Present results of each meta-analysis done, including confidence intervals and measures of consistency. | 7-9 |
| Risk of bias across studies | 22 | Present results of any assessment of risk of bias across studies (see Item 15). | 9-10 |
| Additional analysis | 23 | Give results of additional analyses, if done (e.g., sensitivity or subgroup analyses, meta-regression [see Item 16]). | 10 |
| **DISCUSSION** |  |  |  |
| Summary of evidence | 24 | Summarize the main findings including the strength of evidence for each main outcome; consider their relevance to key groups (e.g., healthcare providers, users, and policy makers). | 10 |
| Limitations | 25 | Discuss limitations at study and outcome level (e.g., risk of bias), and at review-level (e.g., incomplete retrieval of identified research, reporting bias). | 12-13 |
| Conclusions | 26 | Provide a general interpretation of the results in the context of other evidence, and implications for future research. | 13 |
| **FUNDING** |  |  |  |
| Funding | 27 | Describe sources of funding for the systematic review and other support (e.g., supply of data); role of funders for the systematic review. | 14 |

Supplementary Table 2 Search strategy

| **MEDLINE** | |
| --- | --- |
| #1 | "st elevation myocardial infarction"[MeSH Terms] OR “STEMI”[Title/Abstract] OR “myocardial infarction*”[Title/Abstract] OR “ST-segment elevation”[Title/Abstract] |
| #2 | "Percutaneous coronary intervention"[MeSH Terms] OR “Percutaneous coronary intervention*”[Title/Abstract] OR "coronary intervention*"[Title/Abstract] OR "coronary revascularization*"[Title/Abstract] OR "coronary angioplast*"[Title/Abstract] OR "percutaneous transluminal angioplast*"[Title/Abstract] OR "coronary balloon dilation*"[Title/Abstract] OR "coronary balloon angioplast*"[Title/Abstract] OR “primary percutaneous catheterization”[Title/Abstract] OR "myocardial reperfusion"[MeSH Terms] OR "myocardial reperfusion*"[Title/Abstract] OR "myocardial perfusion*"[Title/Abstract] OR "coronary reperfusion*"[Title/Abstract] OR "coronary perfusion*"[Title/Abstract] OR "PCI"[Title/Abstract] OR "PPCI"[Title/Abstract] OR "PTCA"[Title/Abstract] |
| #3 | "intracoronary*"[Title/Abstract] OR "intra-coronar*"[Title/Abstract] |
| #4 | "glycoprotein IIb/IIIa"[Title/Abstract] OR "abciximab"[MeSH Terms] OR "abciximab"[Title/Abstract] OR "ReoPro"[Title/Abstract] OR "Clotinab"[Title/Abstract] OR "eptifibatide"[MeSH Terms] OR "eptifibatide"[Title/Abstract] OR "Integrilin"[Title/Abstract] OR "Integrelin"[Title/Abstract] OR "tirofiban"[MeSH Terms] OR "tirofiban"[Title/Abstract] OR "Aggrastat"[Title/Abstract] OR "Agrastat"[Title/Abstract] OR "cangrelor"[Supplementary Concept] OR "cangrelor"[Title/Abstract] OR "Kengreal"[Title/Abstract] |
| #5 | ("randomized controlled trial"[Publication Type] OR "controlled clinical trial"[Publication Type] OR "clinical trials as topic"[MeSH Terms:noexp] OR "random*"[Title/Abstract] OR "placebo"[Title/Abstract] OR "blind*"[Title/Abstract] OR “assign*"[Title/Abstract] OR “allocat*"[Title/Abstract] OR "trial"[Title]) NOT ("animals"[MeSH Terms] NOT "humans"[MeSH Terms]) |
| #6 | #1 AND #2 AND #3 AND #4 AND #5 |
| **Embase** | |
| #1 | 'st segment elevation myocardial infarction'/exp OR STEMI:ti,ab OR 'myocardial infarction*':ti,ab OR ‘ST-segment elevation’:ti,ab |
| #2 | 'percutaneous coronary intervention'/exp OR 'heart muscle reperfusion'/exp OR ‘percutaneous coronary intervention*’:ti,ab OR ‘coronary intervention*’:ti,ab OR ‘coronary revascularization*’:ti,ab OR ‘coronary angioplast*’:ti,ab OR ‘percutaneous transluminal angioplast*’:ti,ab OR ‘coronary balloon dilation*’:ti,ab OR ‘coronary balloon angioplast*’:ti,ab OR ‘primary percutaneous catheterization*’:ti,ab OR ‘myocardial reperfusion*’:ti,ab OR ‘myocardial perfusion*’:ti,ab OR ‘coronary reperfusion*’:ti,ab OR ‘coronary perfusion*’:ti,ab OR ‘PCI’:ti,ab OR ‘PPCI’:ti,ab OR ‘PTCA’:ti,ab |
| #3 | 'intracoronary*':ti,ab OR 'intra-coronar*':ti,ab |
| #4 | (glycoprotein IIb IIIa):ti,ab,kw OR ‘abciximab’/exp OR ‘eptifibatide’/exp OR ‘tirofiban’/exp OR ‘cangrelor’/exp OR ‘glycoprotein IIb/IIIa’:ti,ab OR ‘abciximab’:ti,ab OR ‘ReoPro’:ti,ab OR ‘Clotinab’:ti,ab OR ‘eptifibatide’:ti,ab OR ‘Integrilin’:ti,ab OR ‘Integrelin’:ti,ab OR ‘tirofiban’:ti,ab OR ‘Aggrastat’:ti,ab OR ‘Agrastat’:ti,ab OR ‘cangrelor’:ti,ab OR ‘Kengreal’:ti,ab |
| #5 | (('crossover procedure':de OR 'double-blind procedure':de OR 'randomized controlled trial':de OR 'single-blind procedure':de OR (random* OR crossover* OR cross NEXT/1 over* OR placebo* OR doubl* NEAR/1 blind* OR singl* NEAR/1 blind* OR assign* OR allocat*):de,ab,ti OR trial:ti) AND [embase]/lim) NOT ([animal cell]/lim OR [animal experiment]/lim OR [animal model]/lim OR [animal tissue]/lim) |
| #6 | #1 AND #2 AND #3 AND #4 AND #5 |
| **CENTRAL** | |
| #1 | MeSH descriptor: [ST Elevation Myocardial Infarction] explode all trees |
| #2 | (STEMI):ti,ab,kw OR (myocardial infarction*):ti,ab,kw OR (ST-segment elevation):ti,ab,kw |
| #3 | #1 OR #2 |
| #4 | MeSH descriptor: [Percutaneous Coronary Intervention] explode all trees |
| #5 | MeSH descriptor: [Myocardial Reperfusion] explode all trees |
| #6 | (percutaneous coronary intervention*):ti,ab,kw OR (coronary intervention*):ti,ab,kw OR (coronary revascularization*):ti,ab,kw OR (coronary angioplast*):ti,ab,kw OR (percutaneous transluminal angioplast*):ti,ab,kw OR (coronary balloon dilation*):ti,ab,kw OR (coronary balloon angioplast*):ti,ab,kw OR (primary percutaneous catheterization*):ti,ab,kw OR (myocardial reperfusion*):ti,ab,kw OR (myocardial perfusion*):ti,ab,kw OR (coronary reperfusion*):ti,ab,kw OR (coronary perfusion*):ti,ab,kw OR PCI:ti,ab,kw OR PPCI:ti,ab,kw OR PTCA:ti,ab,kw |
| #7 | #4 OR #5 OR #6 |
| #8 | intracoronar*:ti,ab,kw OR intra-coronar*:ti,ab,kw |
| #9 | MeSH descriptor: [Abciximab] explode all trees |
| #10 | MeSH descriptor: [Eptifibatide] explode all trees |
| #11 | MeSH descriptor: [Tirofiban] explode all trees |
| #12 | (glycoprotein IIb IIIa):ti,ab,kw OR abciximab:ti,ab,kw OR ReoPro:ti,ab,kw OR Clotinab:ti,ab,kw OR eptifibatide:ti,ab,kw OR Integrilin:ti,ab,kw OR Integrelin:ti,ab,kw OR tirofiban:ti,ab,kw OR Aggrastat:ti,ab,kw OR Agrastat:ti,ab,kw OR cangrelor:ti,ab,kw OR Kengreal:ti,ab,kw |
| #13 | #9 OR #10 OR #11 OR #12 |
| #14 | #3 AND #7 AND #8 AND #13 |

Supplementary Table 3 Baseline patient characteristics

| **First author, year**  **(country)** | **Sample size (IC/IV group)** | **Age, years, mean±SD** | **Men, %** | **Smoking, %** | **Diabetes, %** | **Hypertension, %** | **Dyslipidemia, %** | **Pre-PCI TIMI grade 0-1 flow, %** | **Anterior infarction, %** | **Multivessel involvement**  **(≥ 2 vessels), %** |
| --- | --- | --- | --- | --- | --- | --- | --- | --- | --- | --- |
| Bellandi, 2004  (Italy) | 22 / 23 | 61.9±12.4 | 77.5 | 46.5 | 24.0 | 40.0 | 44.5 | 100.0 | 44.4^b^ | 48.9 |
| Thiele, 2008  (Germany) | 77 / 77 | 63.5±13.3 | 79.5 | 50.0 | 30.0 | 72.0 | 37.5 | 71.4 | 54.5 | 50.0 |
| Dominguez-Rodriguez, 2009  (Spain) | 25 / 25 | 68.0±17.0 | 76.0 | 84.0 | 56.0 | 54.0 | 78.0 | 100 (thrombus score 4-5, 62.0%) | 60.0 ^b^ | 44.0 |
| Bertrand, 2010  (Canada) | 53 / 52 | 59.0±9.0 | 80.0 | 73.5 | 9.0 | 43.5 | 47.5 | 72.4 | 41.0^b^ | NA |
| Gu, 2010  (Netherlands) | 271 / 263 | 64.0±13.0 | 74.0 | 45.5 | 12.0 | 46.5 | 29.0 | 61.0 (thrombus present, 89.7%) | 45.9^b^ | 57.9 |
| Eitel, 2011  (Germany) | 77 / 77 | 63.5±13.3 | 85.0 | 50.0 | 30.0 | 72.0 | 37.5 | 71.4 | 54.5 | 50.0 |
| Iversen, 2011 (30 days)  (Denmark) | 185 / 170 | 62.0±11.0 | 80.6 | 51.0 | 12.6 | 40.0 | 45.9 | 86.0 | 49.0^b^ | 33.2 |
| Iversen, 2011 (1 year)  (Denmark) | 185 / 170 | 62.0±11.0 | 80.6 | 51.0 | 12.6 | 40.0 | 45.9 | 86.0 | 49.0^b^ | 33.2 |
| Kirma, 2012  (Turkey) | 25 / 24 | 56.51±7.8 | 90.0 | 75.7 | 16.4 | 26.4 | 8.0 | 100 | 63.3 | 75.5 |
| Thiele, 2012  (Germany) | 1032 / 1033 | 63.0±13.0 | 75.0 | 44.5 | 20.0 | 70.5 | 41.0 | 73.3 | 48.6 | 52.7 |
| Desch, 2013  (Germany) | 925 / 921 | 63.0±13.0 | 75.0 | 44.5 | 20.0 | 70.5 | 41.0 | 73.3 | 48.6 | 52.7 |
| Eitel, 2013  (Germany) | 394 / 401 | 61.4±14.9 | 76.0 | 46.5 | 20.0 | 70.9 | 38.3 | 69.0 | 48.0 | 47.0 |
| Namazi, 2013  (Iran) | 20 / 20 | 56.2±7.8 | 87.5 | 62.5 | 25.0 | 31.2 | 28.5 | 97.5 (angiographically apparent thrombus, 92.5%) | 52.5^b^ | 70.0 |
| Pellicori, 2013  (Italy) | 38 / 39 | 59 [39-82] ^a^ | 81.4 | 56.6 | 21.5 | 49.5 | 71.0 | NA | 57.1^b^ | NA |
| Secco, 2014  (Italy) | 47 / 42 | 61.4±12.2 | 83.2 | 43.0 | 9.1 | 52.8 | 35.6 | 80.9 | 44.9 | 44.9 |
| Esfandi, 2016  (Iran) | 36 / 38 | 56.9±10.9 | 70.6 | 36.1 | 32.5 | 53.0 | 48.6 | 93.2 | 51.4^b^ | NA |
| Sanati, 2017  (Iran) | 32 / 32 | 59.2±11.6 | 91.0 | 31.0 | 17.0 | 33.0 | 0.0 | 89.1 | NA | 57.8 |
| Bedjaoui, 2019  (Algeria) | 78 / 82 | 59.7±13.6 | 85.6 | 41.8 | 34.1 | 45.2 | 19.0 | 76.3 | 65.0 | 36.3 |
| Nab, 2019  (Egypt) | 50 / 50 | 51.0±8.07 | 75.0 | 60.0 | 37.0 | 75.0 | 30.0 | 93.0 | 100.0^b^ | NA |
| Ma, 2020  (China) | 106 / 102 | 55.2±12.4 | 80.5 | 70.1 | 27.5 | 40.9 | NA | 82.7 | 41.3 | NA |
| Tang, 2022 (DM)  (China) | 100 / 100 | 63.8±5.4 | 68.0 | NA | 100.0 | NA | NA | NA (patients have high thrombus burden) | NA | NA |
| Tang, 2022  (China) | 90 / 90 | 59.6±10.5 | 70.0 | 52.5 | 35.0 | 60.0 | 10.0 | NA (hyperthrombotic burden, 100.0%) | 39.4 | NA |

^a^ Median[range]

^b^ Reported as left anterior descending

Supplementary Table 4 Meta-regression according to baseline patient characteristics

| **Variables** | **Number of studies** | **Beta coefficients (95% CI)** | ***p*-value** |
| --- | --- | --- | --- |
| ***TIMI grade 3 flow*** |  |  |  |
| Smoking | 15 | -0.0007 (-0.0033‒0.0019) | 0.5927 |
| Diabetes | 16 | 0.0034 (0.0004‒0.0064) | **0.0248** |
| Dyslipidemia | 14 | 0.0014 (-0.0015‒0.0042) | 0.3404 |
| Hypertension | 15 | -0.0004 (-0.0025‒0.0018) | 0.7494 |
| Pre-PCI TIMI grade 0-1 flow | 14 | 0.0013 (-0.0012-0.0038) | 0.3045 |
| Anterior infarction | 14 | 0.0001 (-0.0020-0.0022) | 0.9514 |
| Multivessel PCI | 10 | -0.0021 (-0.0057-0.0014) | 0.2408 |
| ***Myocardial blush grade 2/3*** |  |  |  |
| Smoking | 7 | -0.0020 (-0.0110‒0.0070) | 0.6644 |
| Diabetes | 7 | 0.0060 (-0.0064‒0.0184) | 0.3441 |
| Dyslipidemia | 7 | 0.0054 (0.0000‒0.0107) | **0.0483** |
| Hypertension | 7 | 0.0047 (-0.0019‒0.0112) | 0.1665 |
| Pre-PCI TIMI grade 0-1 flow | 7 | 0.0025 (-0.0065-0.0114) | 0.5904 |
| Anterior infarction | 7 | 0.0045 (-0.0035-0.0125) | 0.2715 |
| Multivessel PCI | 5 | -0.0026 (-0.0080-0.0028) | 0.3519 |
| ***Complete ST-segment resolution*** |  |  |  |
| Smoking | 8 | -0.0007 (-0.0070‒0.0056) | 0.8229 |
| Diabetes | 8 | 0.0140 (-0.0045‒0.0072) | 0.6442 |
| Dyslipidemia | 8 | 0.0023 (-0.0036‒0.0083) | 0.4420 |
| Hypertension | 8 | -0.0017 (-0.0076‒0.0042) | 0.5724 |
| Pre-PCI TIMI grade 0-1 flow | 8 | 0.0021 (-0.0032-0.0074) | 0.4400 |
| Anterior infarction | 7 | -0.0016 (-0.0064-0.0032) | 0.5139 |
| Multivessel PCI | 6 | -0.0081 (-0.0223-0.0060) | 0.2589 |
| ***Corrected TIMI frame count*** |  |  |  |
| Smoking | 4 | 0.0042 (-0.0227‒0.0311) | 0.7605 |
| Diabetes | 4 | -0.0240 (-0.0516‒0.0036) | 0.0880 |
| Dyslipidemia | 4 | -0.0089 (-0.0324‒0.0147) | 0.4597 |
| Hypertension | 4 | 0.0033 (-0.0337‒0.0403) | 0.8600 |
| Pre-PCI TIMI grade 0-1 flow | 4 | -0.0252 (-0.0567-0.0064) | 0.1178 |
| Anterior infarction | 4 | 0.0084 (-0.0452-0.0621) | 0.7577 |
| Multivessel PCI | 3 | 0.0032 (-0.0443-0.0507) | 0.8952 |
| ***MACE within 1 month*** |  |  |  |
| Smoking | 6 | -0.0039 (-0.1100‒0.1023) | 0.9430 |
| Diabetes | 6 | -0.0032 (-0.0576‒0.0512) | 0.9085 |
| Dyslipidemia | 6 | -0.0357 (-0.0786‒0.0072) | 0.1028 |
| Hypertension | 6 | 0.0008 (-0.0448‒0.0464) | 0.9733 |
| Pre-PCI TIMI grade 0-1 flow | 5 | -0.0196 (-0.0560-0.0169) | 0.2927 |
| Anterior infarction | 5 | 0.0058 (-0.0260-0.0375) | 0.3557 |
| Multivessel PCI | 3 | 0.0308 (-0.0154-0.0769) | 0.1915 |
| ***MACE within 6-12 months*** |  |  |  |
| Smoking | 4 | 0.0077 (-0.0657‒0.0812) | 0.8363 |
| Diabetes | 4 | -0.0025 (-0.0806‒0.0756) | 0.9507 |
| Dyslipidemia | 3 | -0.0931 (-0.1919‒0.0056) | 0.0645 |
| Hypertension | 4 | -0.0008 (-0.0577‒0.0560) | 0.9767 |
| Pre-PCI TIMI grade 0-1 flow | 4 | -0.0045 (-0.1371-0.1282) | 0.9474 |
| Anterior infarction | 4 | -0.0860 (-0.2036-0.0316) | 0.1518 |
| Multivessel PCI | 3 | 0.0369 (-0.0768-0.1505) | 0.5248 |
| ***All-cause deaths*** |  |  |  |
| Smoking | 7 | -0.0149 (-0.0793-0.0494) | 0.6495 |
| Diabetes | 7 | 0.0100 (-0.0687-0.0887) | 0.8027 |
| Dyslipidemia | 6 | -0.0248 (-0.1089-0.0594) | 0.5639 |
| Hypertension | 7 | 0.0275 (-0.0005-0.0555) | 0.0543 |
| Pre-PCI TIMI grade 0-1 flow | 7 | -0.0261 (-0.0816-0.0293) | 0.3557 |
| Anterior infarction | 6 | -0.0072 (-0.0471-0.0327) | 0.7235 |
| Multivessel PCI | 4 | 0.0555 (-0.0054-0.1163) | 0.0739 |
| ***Cardiac deaths*** |  |  |  |
| Smoking | 5 | -0.0057 (-0.2225‒0.2111) | 0.9588 |
| Diabetes | 6 | -0.0153 (-0.0415‒0.0110) | 0.2534 |
| Dyslipidemia | 5 | 0.0073 (-0.0695‒0.0842) | 0.8515 |
| Hypertension | 5 | 0.0160 (-0.0351‒0.0671) | 0.5396 |
| Pre-PCI TIMI grade 0-1 flow | 4 | 0.0509 (-0.0492-0.1511) | 0.3187 |
| Anterior infarction | 5 | 0.0714 (-0.0794-0.2223) | 0.3534 |
| Multivessel PCI | 4 | -0.0718 (-0.2484-0.1048) | 0.4257 |
| ***Major bleeding*** |  |  |  |
| Smoking | 9 | -0.0384 (-0.1008‒0.0241) | 0.2284 |
| Diabetes | 9 | 0.0111 (-0.0551‒0.0773) | 0.7434 |
| Dyslipidemia | 8 | 0.0021 (-0.0663‒0.0705) | 0.9524 |
| Hypertension | 9 | 0.0187 (-0.0143‒0.0517) | 0.2671 |
| Pre-PCI TIMI grade 0-1 flow | 9 | -0.0248 (-0.0783-0.0287) | 0.3634 |
| Anterior infarction | 9 | -0.0047 (-0.0598-0.0504) | 0.8673 |
| Multivessel PCI | 6 | 0.0200 (-0.0422-0.0822) | 0.5290 |
| ***Minor bleeding*** |  |  |  |
| Smoking | 8 | 0.0011 (-0.0631‒0.0653) | 0.9738 |
| Diabetes | 8 | 0.0092 (-0.0285‒0.0469) | 0.6319 |
| Dyslipidemia | 8 | -0.0181 (-0.0482‒0.0120) | 0.2381 |
| Hypertension | 8 | 0.0146 (-0.0138‒0.0431) | 0.3136 |
| Pre-PCI TIMI grade 0-1 flow | 7 | -0.0213 (-0.0575-0.0149) | 0.2497 |
| Anterior infarction | 7 | 0.0034 (-0.0227-0.0296) | 0.7972 |
| Multivessel PCI | 5 | 0.0421 (0.0087-0.0754) | **0.0134** |

Note: Significance level < 0.05 (in bold**)**.

Abbreviations: CI, confidence interval; MACE, major cardiac adverse events; PCI, Percutaneous coronary intervention.

Supplementary Table 5 Detailed information regarding the risk of bias assessment according to the Risk of Bias 2 criteria

| **First author, year** | **The risk of bias domain** | **Assessment of risk of bias** | **The reason for judgment** |
| --- | --- | --- | --- |
| Bellandi, 2004 | Randomization process | Some concerns | Although authors mentioned that randomization was conducted, the specific method was not described.  There was no substantial baseline imbalance between the two groups. |
|  | Deviations from intended interventions | Low risk | Operator physicians were aware of intervention group. There was no deviation from the intended intervention because of the trial context. In addition, modified intention-to-treat analysis was done. |
|  | Missing outcome data | Low risk | Outcome data were available for all participants, except LVEF. Missing data for LVEF were balanced between the two groups. |
|  | Measurement of the outcome | Low risk | The method of measuring outcomes was appropriate and did not differ between the two groups. The outcome assessors were blinded. |
|  | Selection of the reported result | Low risk | A pre-existing protocol was described, and all the results were reported in the pre-specified manner. |
|  | Overall | Some concerns |  |
| Thiele, 2008 | Randomization process | Low risk | Balanced randomization was performed by drawing sealed unlabeled envelopes placed in no order in an urn. There was no substantial baseline imbalance between the two groups. |
|  | Deviations from intended interventions | Low risk | Operator physicians and participants were aware of intervention group. All patients received the assigned treatment. |
|  | Missing outcome data | Low risk | Outcome data were available for all participants, except LVEF. Missing data for LVEF were balanced between the two groups. |
|  | Measurement of the outcome | Low risk | The method of measuring outcomes was appropriate and did not differ between the two groups. The outcome assessors were blinded. |
|  | Selection of the reported result | Low risk | A pre-existing protocol was described, and all the results were reported in the pre-specified manner. |
|  | Overall | Low risk |  |
| Dominguez-Rodriguez, 2009 | Randomization process | Some concerns | Although authors mentioned that randomization was conducted, the specific method was not described.  There was no substantial baseline imbalance between the two groups. |
|  | Deviations from intended interventions | Low risk | Operator physicians and participants were aware of intervention group. There was probably no deviation from the intended intervention because of the trial context. |
|  | Missing outcome data | Low risk | Outcome data were available for all participants. |
|  | Measurement of the outcome | Low risk | The method of measuring outcomes was appropriate and did not differ between the two groups. The outcome assessors were blinded. |
|  | Selection of the reported result | Low risk | A pre-existing protocol was described, and all the results were reported in the pre-specified manner. |
|  | Overall | Some concerns |  |
| Bertrand, 2010 | Randomization process | Low risk | Randomization was done by means of sealed envelopes. There was no substantial baseline imbalance between the two groups. |
|  | Deviations from intended interventions | Low risk | Authors mentioned that the trial was double-blind. Volume matched placebo was used. There was probably no deviation from the intended intervention because of the trial context. |
|  | Missing outcome data | Low risk | Outcome data were available for nearly all participants. Missing data were balanced between the two groups. |
|  | Measurement of the outcome | Low risk | The method of measuring outcomes was appropriate and did not differ between the two groups. The outcome assessors were blinded. |
|  | Selection of the reported result | Low risk | A pre-existing protocol was described, and all the results were reported in the pre-specified manner. |
|  | Overall | Low risk |  |
| Gu, 2010 | Randomization process | Low risk | Randomization was done by means of sealed envelopes. There was no substantial baseline imbalance between the two groups. |
|  | Deviations from intended interventions | Low risk | Operator physicians were aware of intervention group. There was probably no deviation from the intended intervention because of the trial context. Crossovers were low and unintentionally. |
|  | Missing outcome data | Low risk | There were no significant differences between the drop-out rates or reasons for drop-out between the two groups, and sensitivity analyses were also performed. |
|  | Measurement of the outcome | Low risk | The method of measuring outcomes was appropriate and did not differ between the two groups. The outcome assessors were blinded. |
|  | Selection of the reported result | Low risk | A pre-existing protocol was described, and all the results were reported in the pre-specified manner. |
|  | Overall | Low risk |  |
| Eitel, 2011 | Randomization process | Low risk | Balanced randomization was performed by drawing sealed unlabeled envelopes placed in no order in an urn. There was no substantial baseline imbalance between the two groups. |
|  | Deviations from intended interventions | Low risk | Operator physicians and participants were aware of intervention group. All patients received the assigned treatment. |
|  | Missing outcome data | Low risk | Outcome data were available for all participants, except LVEF. Missing data for LVEF were balanced between the two groups. |
|  | Measurement of the outcome | Low risk | The method of measuring outcomes was appropriate and did not differ between the two groups. The outcome assessors were blinded. |
|  | Selection of the reported result | Low risk | A pre-existing protocol was described, and all the results were reported in the pre-specified manner. |
|  | Overall | Low risk |  |
| Iversen, 2011 (30 days) | Randomization process | Low risk | Randomization was done by means of sealed opaque envelopes. There was no substantial baseline imbalance between the two groups. |
|  | Deviations from intended interventions | Low risk | Operator physicians and participants were aware of intervention group. There was probably no deviation from the intended intervention because of the trial context. |
|  | Missing outcome data | Low risk | Outcome data were available for all participants. |
|  | Measurement of the outcome | Low risk | The method of measuring outcomes was appropriate and did not differ between the two groups. The outcome assessors were blinded. |
|  | Selection of the reported result | Low risk | A pre-existing protocol was described, and all the results were reported in the pre-specified manner. |
|  | Overall | Low risk |  |
| Iversen, 2011 (1 year) | Randomization process | Low risk | Randomization was done by means of sealed opaque envelopes. There was no substantial baseline imbalance between the two groups. |
|  | Deviations from intended interventions | Low risk | Operator physicians and participants were aware of intervention group. There was probably no deviation from the intended intervention because of the trial context. |
|  | Missing outcome data | Low risk | Outcome data were available for all participants. |
|  | Measurement of the outcome | Low risk | The method of measuring outcomes was appropriate and did not differ between the two groups. The outcome assessors were blinded. |
|  | Selection of the reported result | Low risk | A pre-existing protocol was described, and all the results were reported in the pre-specified manner. |
|  | Overall | Low risk |  |
| Kirma, 2012 | Randomization process | Low risk | Balanced randomization was performed by drawing sealed unlabeled envelopes placed in random order. |
|  | Deviations from intended interventions | Low risk | Operator physicians and participants were aware of intervention group (open-label). There was probably no deviation from the intended intervention because of the trial context. |
|  | Missing outcome data | Low risk | There were no significant differences between the drop-out rates or reasons for drop-out between the two groups, and sensitivity analyses were also performed. |
|  | Measurement of the outcome | Low risk | The method of measuring outcomes was appropriate and did not differ between the two groups. The outcome assessors were blinded. |
|  | Selection of the reported result | Low risk | A pre-existing protocol was described, and all the results were reported in the pre-specified manner. |
|  | Overall | Low risk |  |
| Thiele, 2012 | Randomization process | Low risk | Randomization was done centrally by web-based program. There was no substantial baseline imbalance between groups. |
|  | Deviations from intended interventions | Low risk | Although operator physicians and participants were aware of intervention group, there was no deviation from the intended intervention because of the trial context. Post-randomization exclusions of ineligible participants is considered appropriate. Crossovers were low. In addition, modified intention-to-treat analysis was done. |
|  | Missing outcome data | Low risk | There were no significant differences between the drop-out rates or reasons for drop-out between the two groups, and sensitivity analyses were also performed. |
|  | Measurement of the outcome | Low risk | The method of measuring outcomes was appropriate and did not differ between the two groups. The outcome assessors were blinded. |
|  | Selection of the reported result | Low risk | A pre-existing protocol was described, and all the results were reported in the pre-specified manner. |
|  | Overall | Low risk |  |
| Desch, 2013 | Randomization process | Low risk | Randomization was done centrally by web-based program. There was no substantial baseline imbalance between groups. |
|  | Deviations from intended interventions | Low risk | Although operator physicians and participants were aware of intervention group, there was no deviation from the intended intervention because of the trial context. |
|  | Missing outcome data | Low risk | Outcome data were available for nearly all participants. Missing data were balanced between the two groups. |
|  | Measurement of the outcome | Low risk | The method of measuring outcomes was appropriate and did not differ between the two groups. The outcome assessors were blinded. |
|  | Selection of the reported result | Low risk | A pre-existing protocol was described, and all the results were reported in the pre-specified manner. |
|  | Overall | Low risk |  |
| Eitel, 2013 | Randomization process | Low risk | Randomization was done centrally by web-based program. There was no substantial baseline imbalance between groups. |
|  | Deviations from intended interventions | Low risk | Although operator physicians and participants were aware of intervention group, there was no deviation from the intended intervention because of the trial context. |
|  | Missing outcome data | Low risk | There were no significant differences between the drop-out rates or reasons for drop-out between the two groups. |
|  | Measurement of the outcome | Low risk | The method of measuring outcomes was appropriate and did not differ between the two groups. The outcome assessors were blinded. |
|  | Selection of the reported result | Low risk | A pre-existing protocol was described, and all the results were reported in the pre-specified manner. |
|  | Overall | Low risk |  |
| Namazi, 2013 | Randomization process | Some concerns | Randomization was done by using random number table. The method of allocation concealment was not described. There was no substantial baseline imbalance between the two groups. |
|  | Deviations from intended interventions | Low risk | Operator physicians were aware of intervention group. There was probably no deviation from the intended intervention because of the trial context. |
|  | Missing outcome data | Low risk | Outcome data were available for all participants. |
|  | Measurement of the outcome | Low risk | The method of measuring outcomes was appropriate and did not differ between the two groups. The outcome assessors were blinded. |
|  | Selection of the reported result | Low risk | A pre-existing protocol was described, and all the results were reported in the pre-specified manner. |
|  | Overall | Some concerns |  |
| Pellicori, 2013 | Randomization process | Some concerns | Although authors mentioned that randomization was conducted, the specific method was not described.  There was no substantial baseline imbalance between the two groups. |
|  | Deviations from intended interventions | Low risk | There was no information about blinding as well as deviation. There was probably no deviation from the intended intervention because of the trial context. |
|  | Missing outcome data | Low risk | There were no significant differences between the drop-out rates or reasons for drop-out between the two groups. |
|  | Measurement of the outcome | Low risk | The method of measuring outcomes was appropriate and did not differ between the two groups. The outcome assessors were blinded. |
|  | Selection of the reported result | Low risk | A pre-existing protocol was described, and all the results were reported in the pre-specified manner. |
|  | Overall | Some concerns |  |
| Secco, 2014 | Randomization process | Low risk | Randomization was done by means of sealed envelopes. There was no substantial baseline imbalance between the two groups, although sample sizes were unequal. |
|  | Deviations from intended interventions | Low risk | There was no information about blinding as well as deviation. There was probably no deviation from the intended intervention because of the trial context. |
|  | Missing outcome data | Low risk | Outcome data were available for all participants. |
|  | Measurement of the outcome | Low risk | The method of measuring outcomes was appropriate and did not differ between the two groups. The outcome assessors were blinded. |
|  | Selection of the reported result | Low risk | A pre-existing protocol was described, and all the results were reported in the pre-specified manner. |
|  | Overall | Low risk |  |
| Esfandi, 2016 | Randomization process | Some concerns | The simple randomization by the online website, http://www.randomizer.org, was used. The method of allocation concealment was not described. There was no substantial baseline imbalance between the two groups. |
|  | Deviations from intended interventions | Low risk | Operator physicians were aware of intervention group (single-blind). There was probably no deviation from the intended intervention because of the trial context. |
|  | Missing outcome data | Low risk | Outcome data were available for nearly all participants. Missing data were balanced between the two groups. |
|  | Measurement of the outcome | Low risk | The method of measuring outcomes was appropriate and did not differ between the two groups. The outcome assessors were blinded. |
|  | Selection of the reported result | Low risk | A pre-existing protocol was described, and all the results were reported in the pre-specified manner. |
|  | Overall | Some concerns |  |
| Sanati, 2017 | Randomization process | Low risk | Randomization was done by computerized balanced block randomization method and by means of sealed envelopes. There was no substantial baseline imbalance between the two groups. |
|  | Deviations from intended interventions | Low risk | There was no information about blinding as well as deviation. There was probably no deviation from the intended intervention because of the trial context. |
|  | Missing outcome data | Low risk | Outcome data were available for nearly all participants. Missing data were balanced between the two groups. |
|  | Measurement of the outcome | Low risk | The method of measuring outcomes was appropriate and did not differ between the two groups. Although there was no information on whether assessors were blinded, assessment was unlikely to be influenced. |
|  | Selection of the reported result | Low risk | A pre-existing protocol was described, and all the results were reported in the pre-specified manner. |
|  | Overall | Low risk |  |
| Bedjaoui, 2019 | Randomization process | Some concerns | Although authors mentioned that randomization was conducted, the specific method was not described.  There was no substantial baseline imbalance between the two groups. |
|  | Deviations from intended interventions | Low risk | Operator physicians were aware of intervention group (single-blind). There was probably no deviation from the intended intervention because of the trial context. |
|  | Missing outcome data | Low risk | Outcome data were available for nearly all participants. Missing data were balanced between the two groups. |
|  | Measurement of the outcome | Low risk | The method of measuring outcomes was appropriate and did not differ between the two groups. Although there was no information on whether assessors were blinded, assessment was unlikely to be influenced. |
|  | Selection of the reported result | Low risk | A pre-existing protocol was described, and all the results were reported in the pre-specified manner. |
|  | Overall | Some concerns |  |
| Nab, 2019 | Randomization process | Some concerns | Computer based randomization was done. The method of allocation concealment was not described. There was no substantial baseline imbalance between the two groups. |
|  | Deviations from intended interventions | Low risk | There was no information about blinding as well as deviation. There was probably no deviation from the intended intervention because of the trial context. |
|  | Missing outcome data | Low risk | Outcome data were available for all participants. |
|  | Measurement of the outcome | Low risk | The method of measuring outcomes was appropriate and did not differ between the two groups. The outcome assessors were blinded. |
|  | Selection of the reported result | Low risk | A pre-existing protocol was described, and all the results were reported in the pre-specified manner. |
|  | Overall | Some concerns |  |
| Ma, 2020 | Randomization process | Some concerns | Although authors mentioned that randomization was conducted, the specific method was not described.  There was no substantial baseline imbalance between the two groups. |
|  | Deviations from intended interventions | Low risk | Operator physicians were aware of intervention group, however there was no deviation from the intended intervention because of the trial context. |
|  | Missing outcome data | Low risk | Missing data were balanced between the two groups |
|  | Measurement of the outcome | Low risk | The method of measuring outcomes was appropriate and did not differ between the two groups. The outcome assessors were blinded. |
|  | Selection of the reported result | Low risk | A pre-existing protocol was described, and all the results were reported in the pre-specified manner. |
|  | Overall | Some concerns |  |
| Tang, 2022 (DM) | Randomization process | Some concerns | Although authors mentioned that randomization was conducted, the specific method was not described.  There was no information about baseline characteristics except sex and age. |
|  | Deviations from intended interventions | Low risk | There was no information about blinding as well as deviation. There was probably no deviation from the intended intervention because of the trial context. |
|  | Missing outcome data | Low risk | Outcome data were available for all participants. |
|  | Measurement of the outcome | Low risk | The method of measuring outcomes was appropriate and did not differ between the two groups. |
|  | Selection of the reported result | Low risk | A pre-existing protocol was described, and all the results were reported in the pre-specified manner. |
|  | Overall | Some concerns |  |
| Tang, 2022 | Randomization process | Some concerns | Randomization was done by using a table of random numbers. The method of allocation concealment was not described. There was no substantial baseline imbalance between groups. |
|  | Deviations from intended interventions | Low risk | Operator physicians were aware of intervention group (single-blind), however there was no deviation from the intended intervention because of the trial context. |
|  | Missing outcome data | Low risk | Outcome data were available for nearly all participants. Missing data were balanced between the two groups |
|  | Measurement of the outcome | Low risk | The method of measuring outcomes was appropriate and did not differ between the two groups. The outcome assessors were blinded. |
|  | Selection of the reported result | Low risk | A pre-existing protocol was described, and all the results were reported in the pre-specified manner. |
|  | Overall | Some concerns |  |

Abbreviation: LVEF, left ventricular ejection fraction.

Supplementary Table 6 Sensitivity analysis based on quality assessment

| **Characteristics** | **Overall studies** | | | **Studies with low risk of bias** | | |
| --- | --- | --- | --- | --- | --- | --- |
|  | **Number of studies**  **(sample size)** | **Pooled RRs (95% CI)** | ***I^2^*** | **Number of studies**  **(sample size)** | **Pooled RRs (95% CI)** | ***I^2^*** |
| **TIMI grade 3 flow** | 16 (4,278) | 1.04 (1.01–1.06) | 45 | 7 (3,221) | 1.01 (0.99–1.04) | 0 |
| **cTFC** | 4 (239) | SMD -0.13 (-0.46–0.20) | 39 | 2 (138) | SMD 0.12 (-0.22–0.45) | 0 |
| **MBG 2/3** | 7 (1,135) | 1.14 (1.07–1.21) | 55 | 4 (830) | 1.13 (1.05–1.22) | 1 |
| **Complete STR** | 8 (1,021) | 1.10 (1.00–1.20) | 0 | 2 (552) | 1.02 (0.90–1.16) | 0 |
| **LVEF after 1 month** | 2 (140) | SMD 0.71 (0.37–1.06) | 0 | - | - | - |
| **LVEF after 6 months or longer** | 3 (302) | SMD 0.25 (0.03–0.48) | 21 | - | - | - |
| **MACE within 1 months** | 6 (1,397) | 0.54 (0.37–0.80) | 0 | 3 (1,043) | 0.53 (0.28–0.98) | 51 |
| **MACE after 6-12 months** | 4 (806) | 0.80 (0.44–1.45) | 64 | 3 (598) | 0.72 (0.35–1.46) | 72 |
| **All-cause death** | 7 (3,181) | 0.88 (0.64–1.21) | 33 | 4 (2,799) | 0.72 (0.35–1.46) | 63 |
| **Cardiac death** | 6 (3,024) | 1.07 (0.73–1.57) | 0 | 4 (2,644) | 1.12 (0.76–1.66) | 0 |
| **Major bleeding** | 9 (3,689) | 1.10 (0.73–1.64) | 0 | 6 (3,221) | 1.16 (0.76–1.77) | 0 |
| **Minor bleeding** | 8 (3,427) | 0.82 (0.62–1.06) | 30 | 3 (2,837) | 0.76 (0.44–1.31) | 75 |

Abbreviations: CI, confidence interval; cTFC, corrected TIMI frame count; LVEF, left ventricular ejection fraction; MACE, major cardiac adverse events; MBG, myocardial blush grade; RRs, risk ratios; SMD, standardized mean difference; STR, ST-segment resolution.


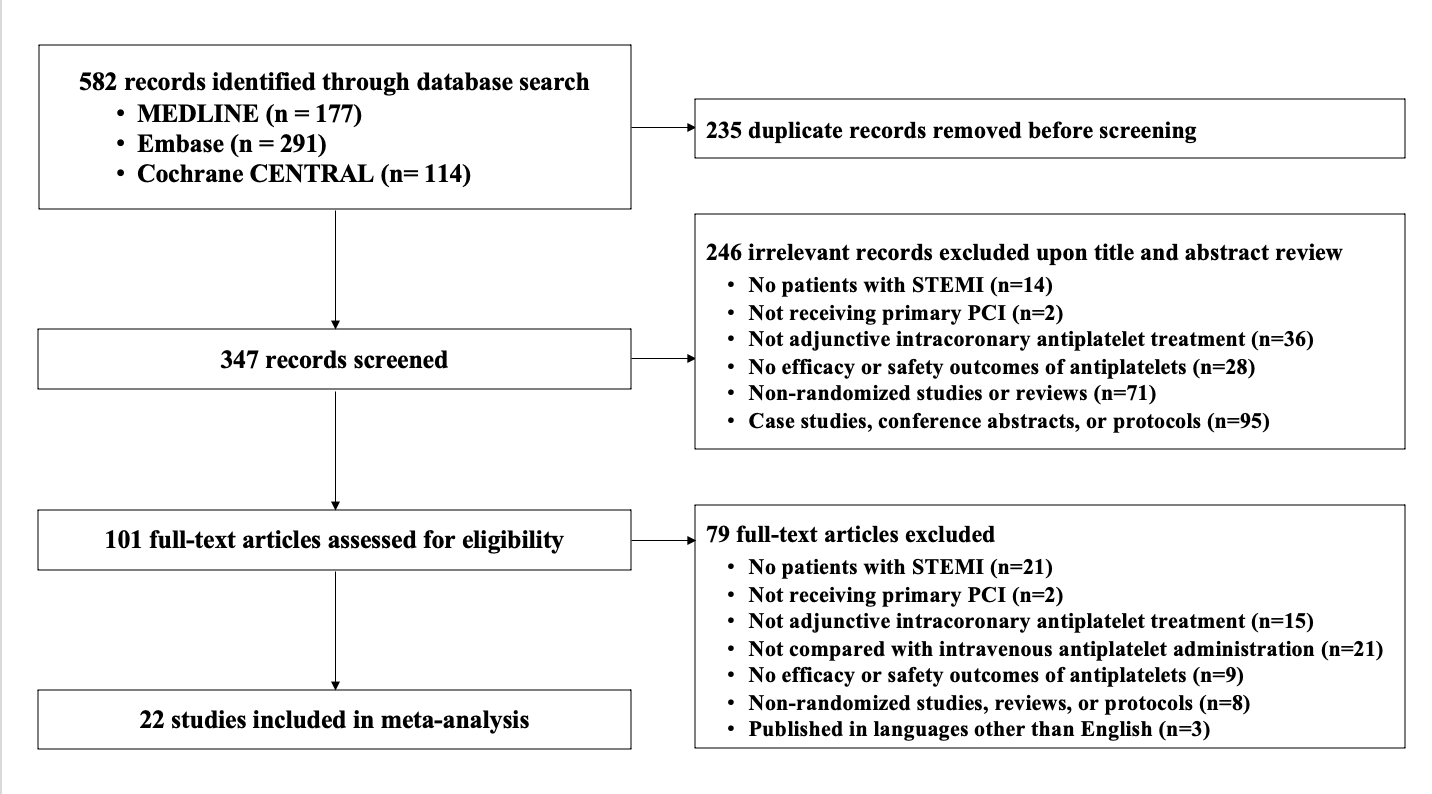


Supplementary Figure 1 Flowchart of the study selection process

**
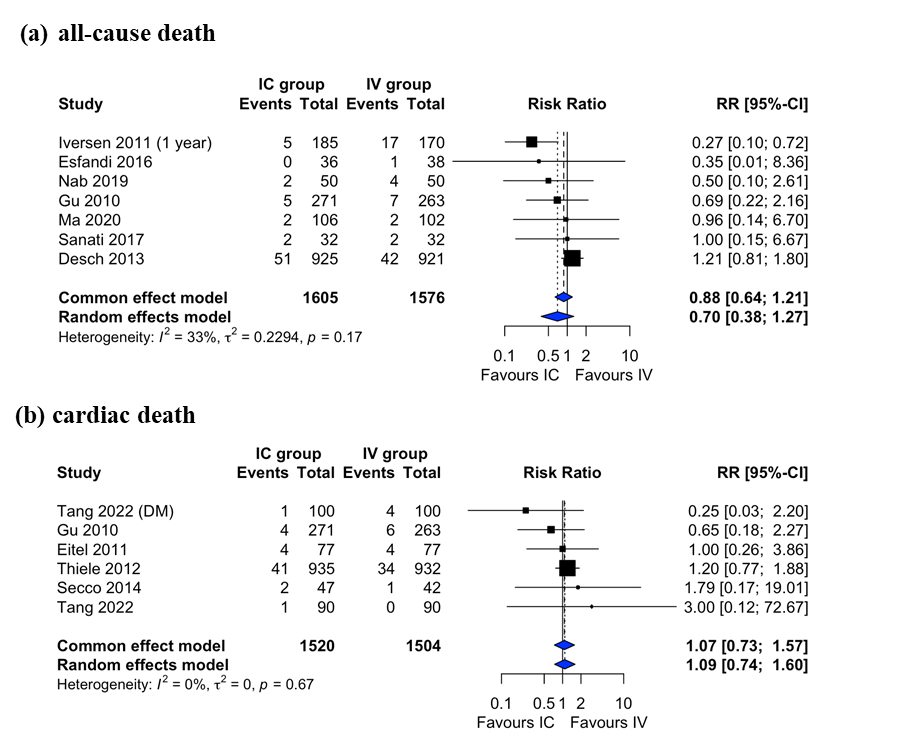
**

Supplementary Figure 2 Forest plot of mortality risk in intracoronary administration of glycoprotein IIb/IIIa inhibitors compared with that in intravenous administration. (a) all-cause death and (b) cardiac death

**
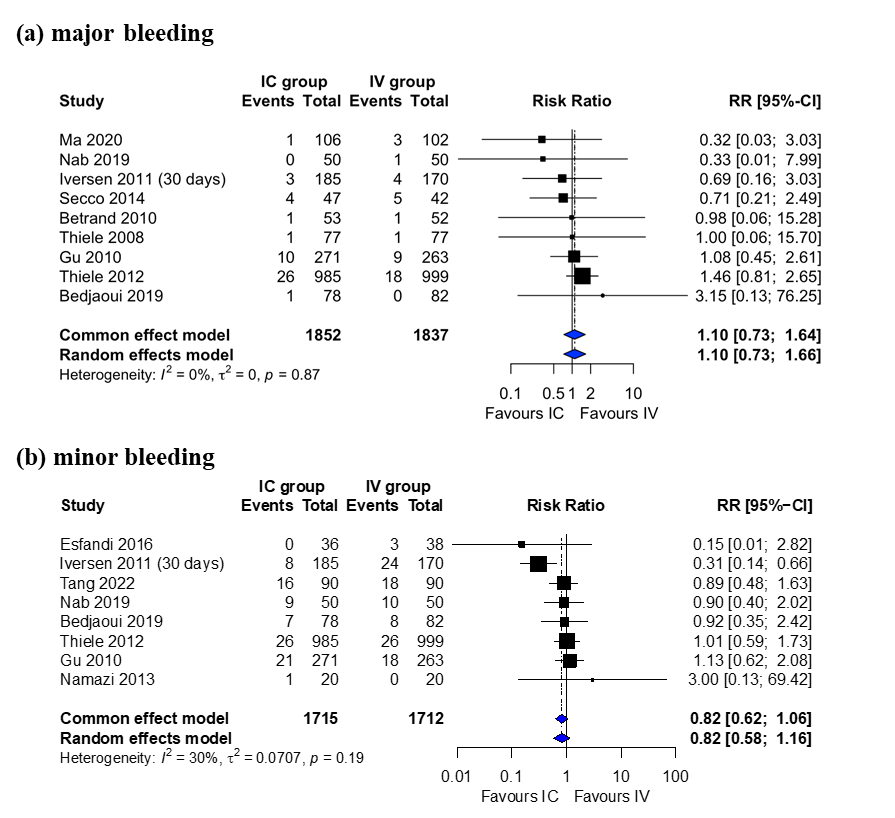
**

Supplementary Figure 3 Forest plot of bleeding risk after intracoronary administration of glycoprotein IIb/IIIa inhibitors compared with that after intravenous administration. (a) major bleeding and (b) minor bleeding

**
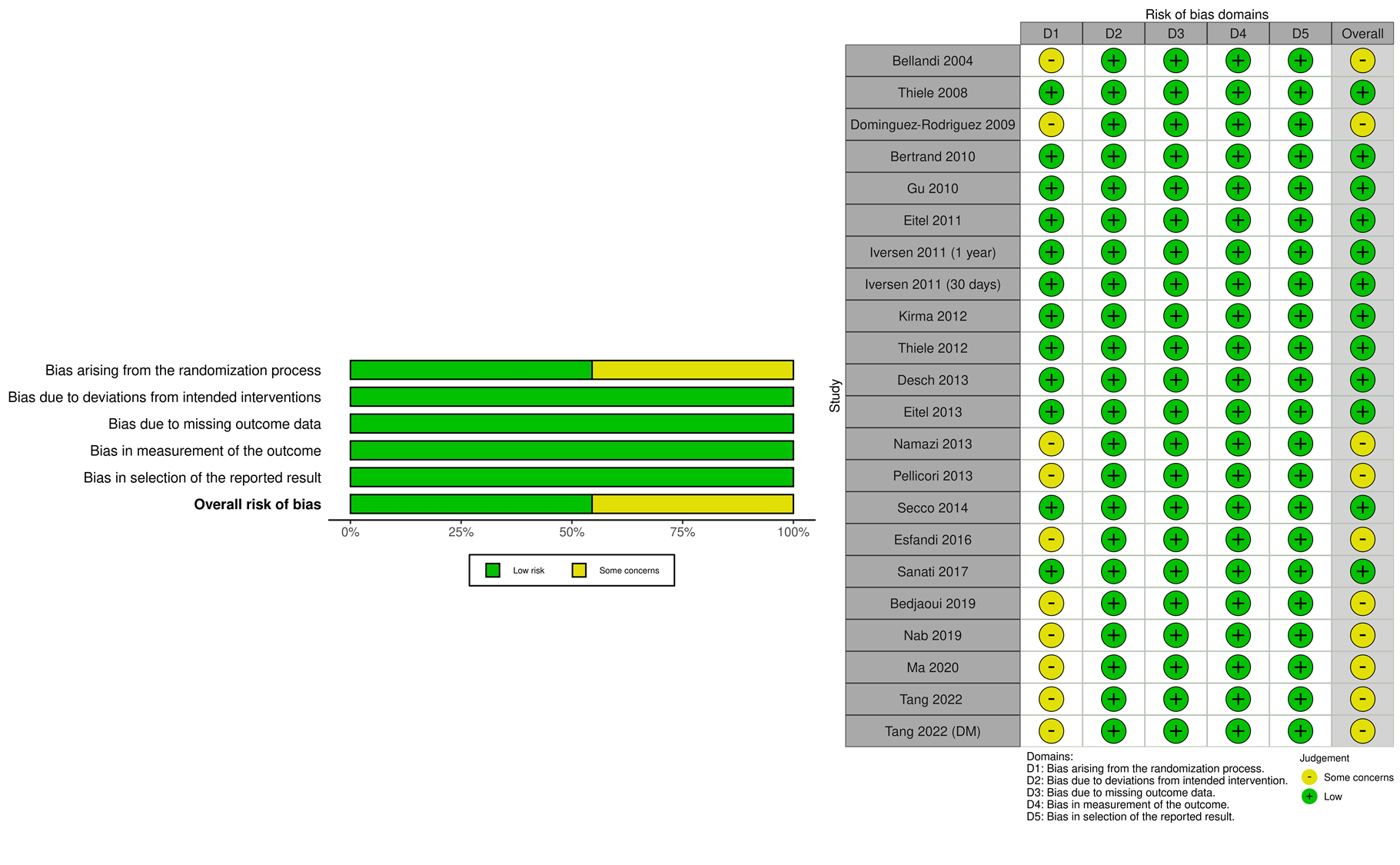
**

Supplementary Figure 4 Quality assessment of the risk of bias according to the Risk of Bias 2 criteria

**
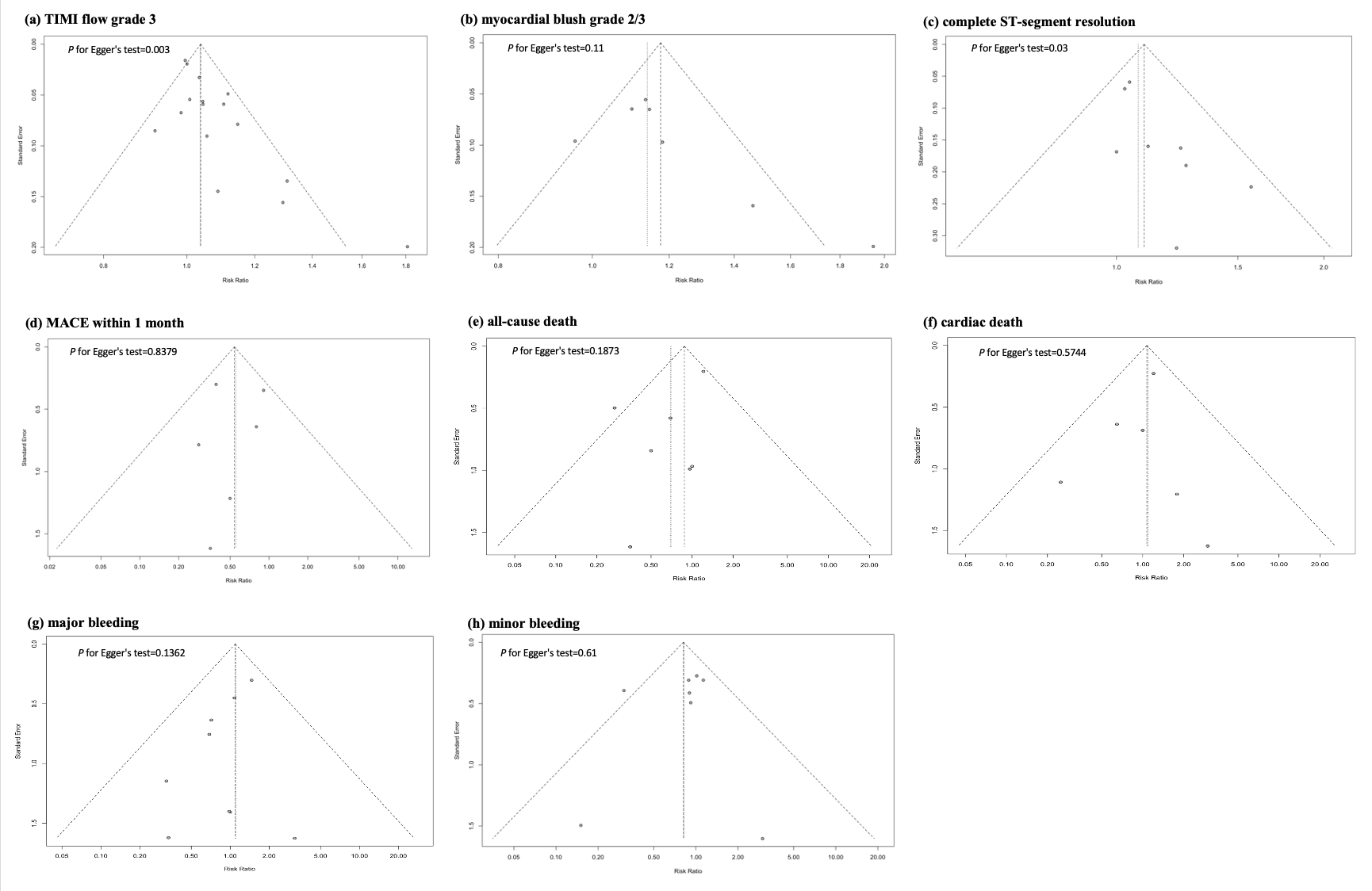
**

Supplementary Figure 5 Funnel plot and Egger’s test for the incidence of myocardial reperfusion and clinical outcomes


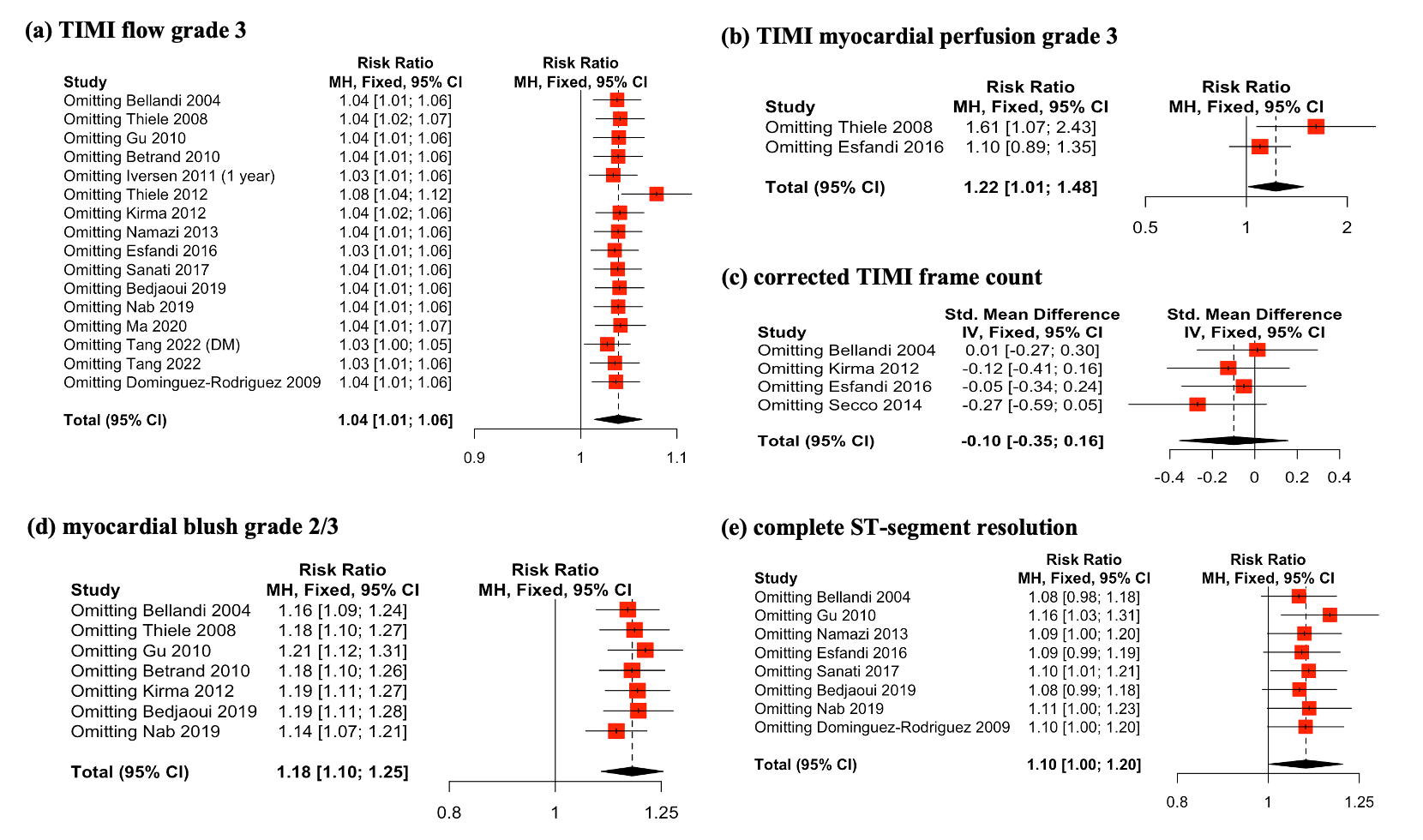


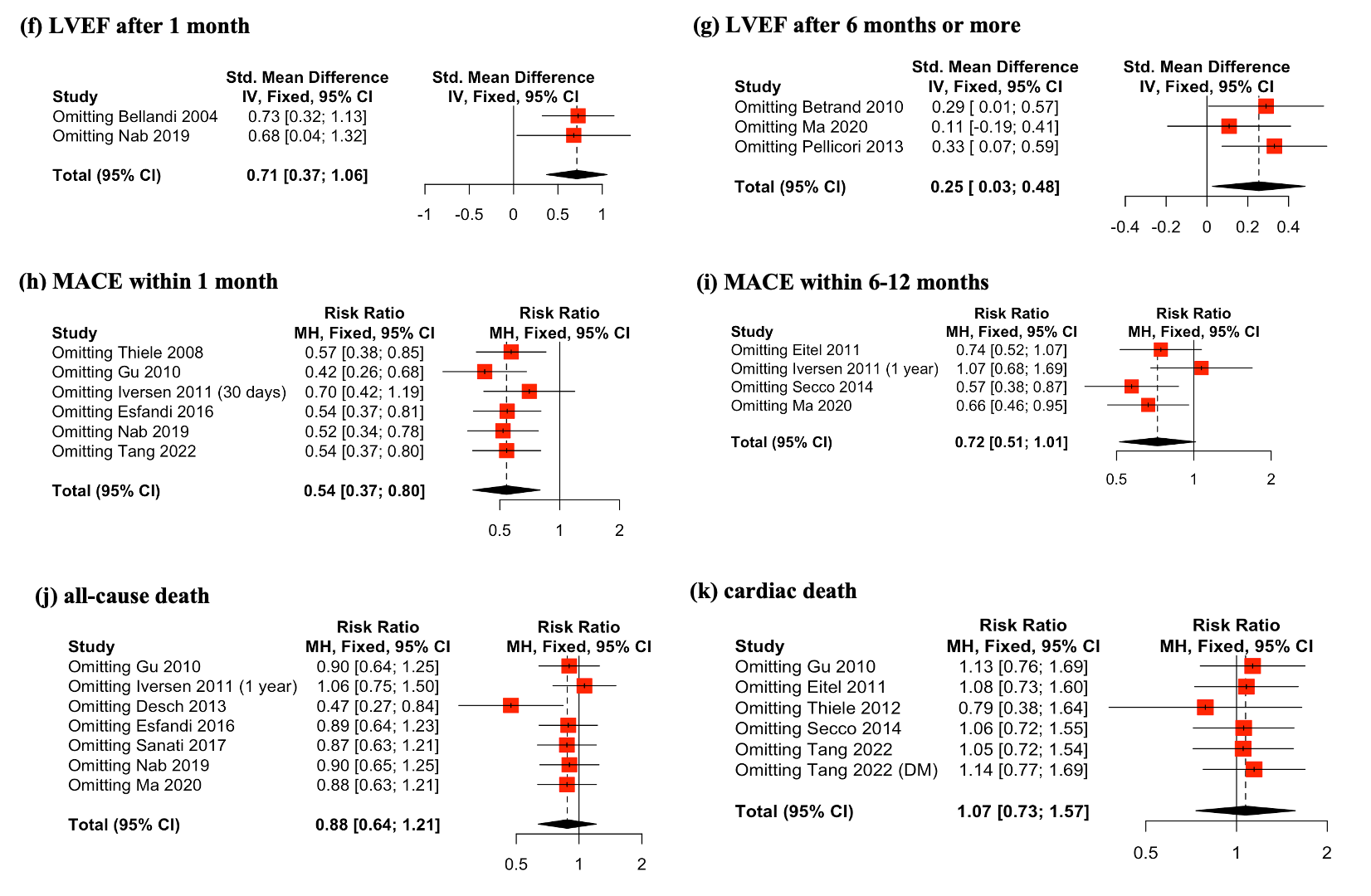


Supplementary Figure 6 Sensitivity analysis with the leave-one-out method

**
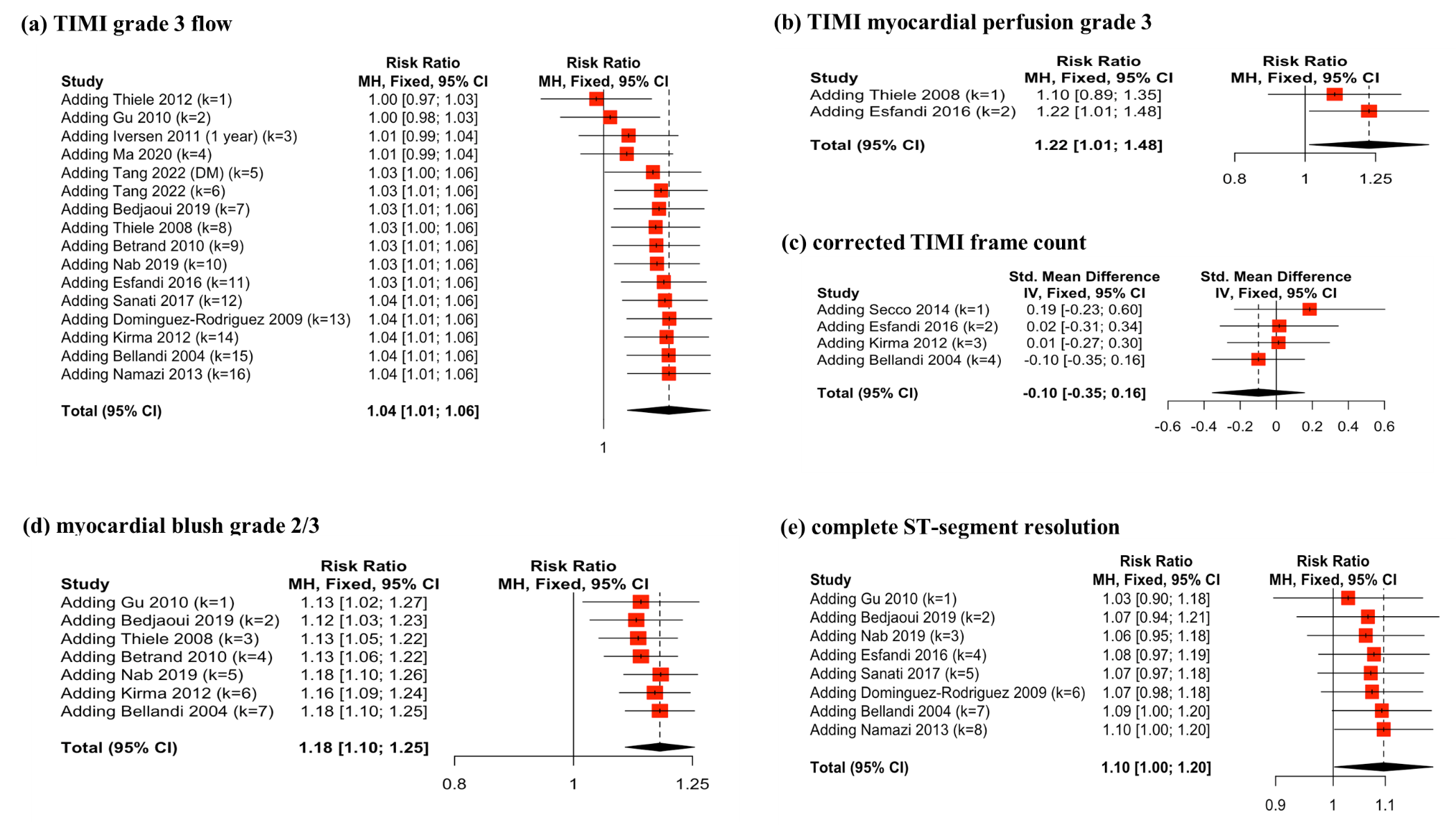
**

**
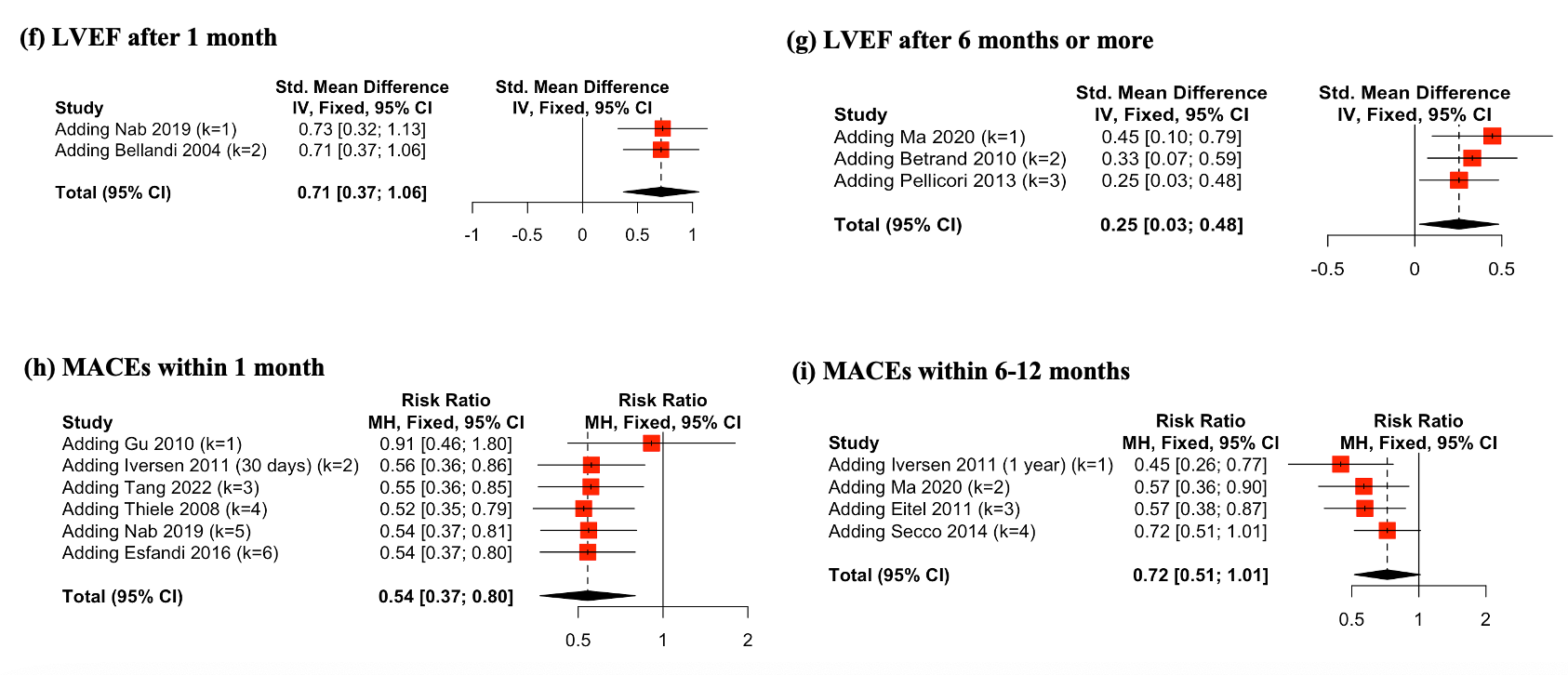
**

**
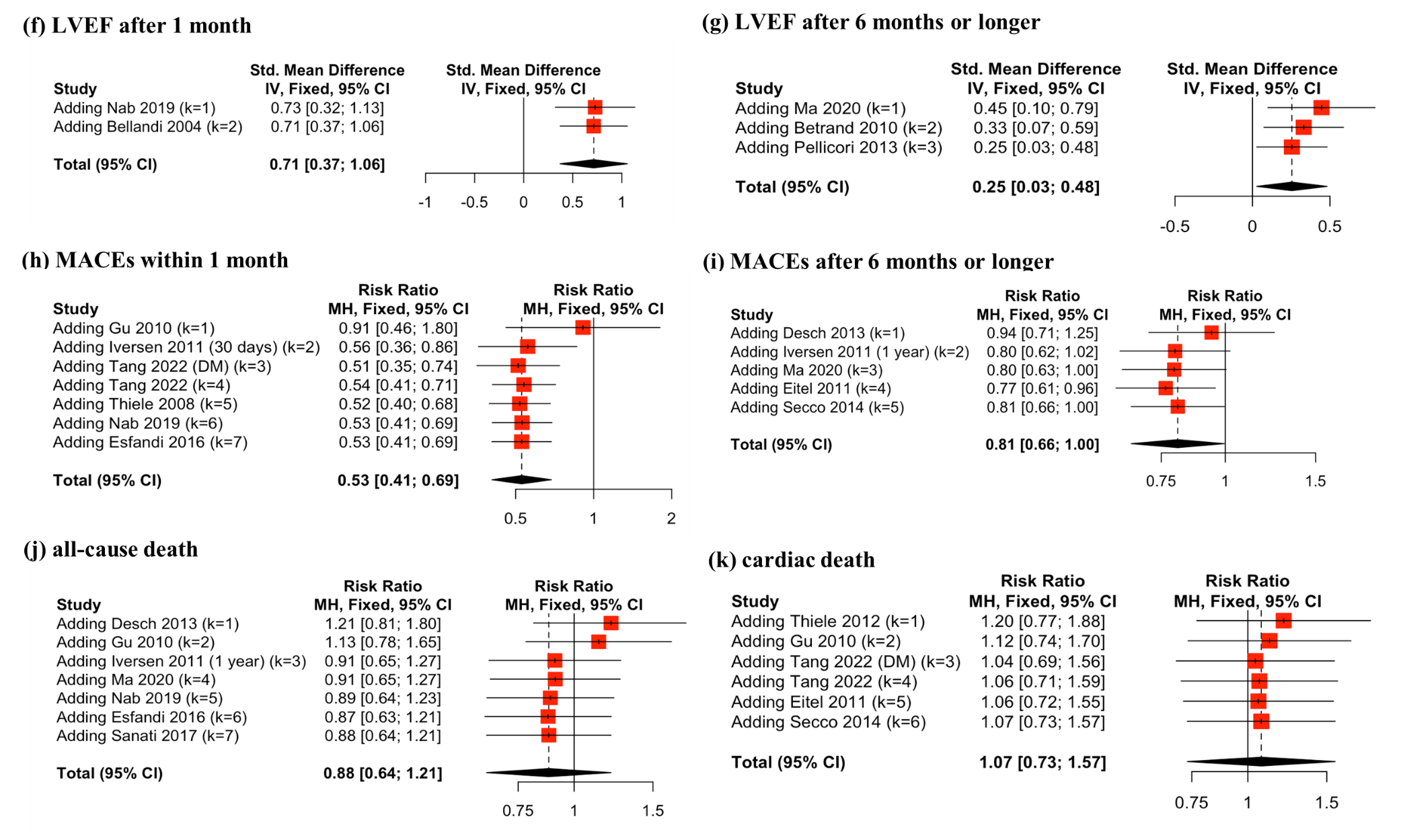
**

**
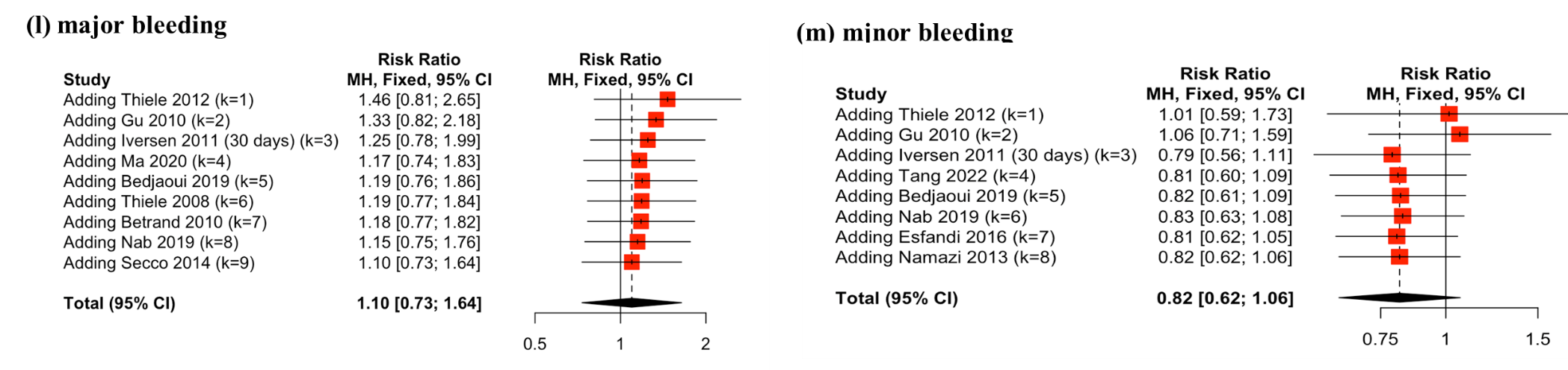
**

Supplementary Figure 7 Sensitivity analysis with a small sample size


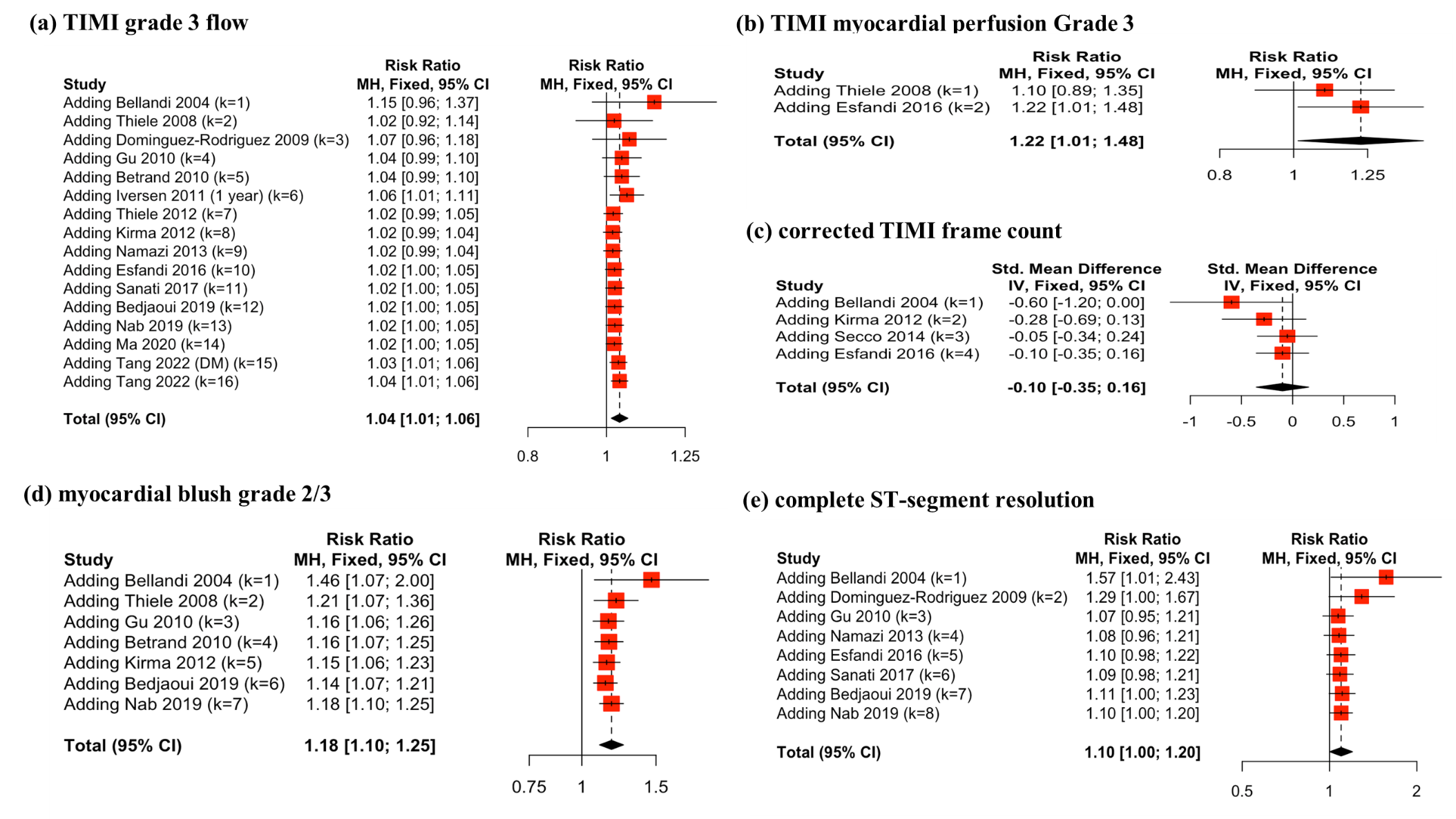


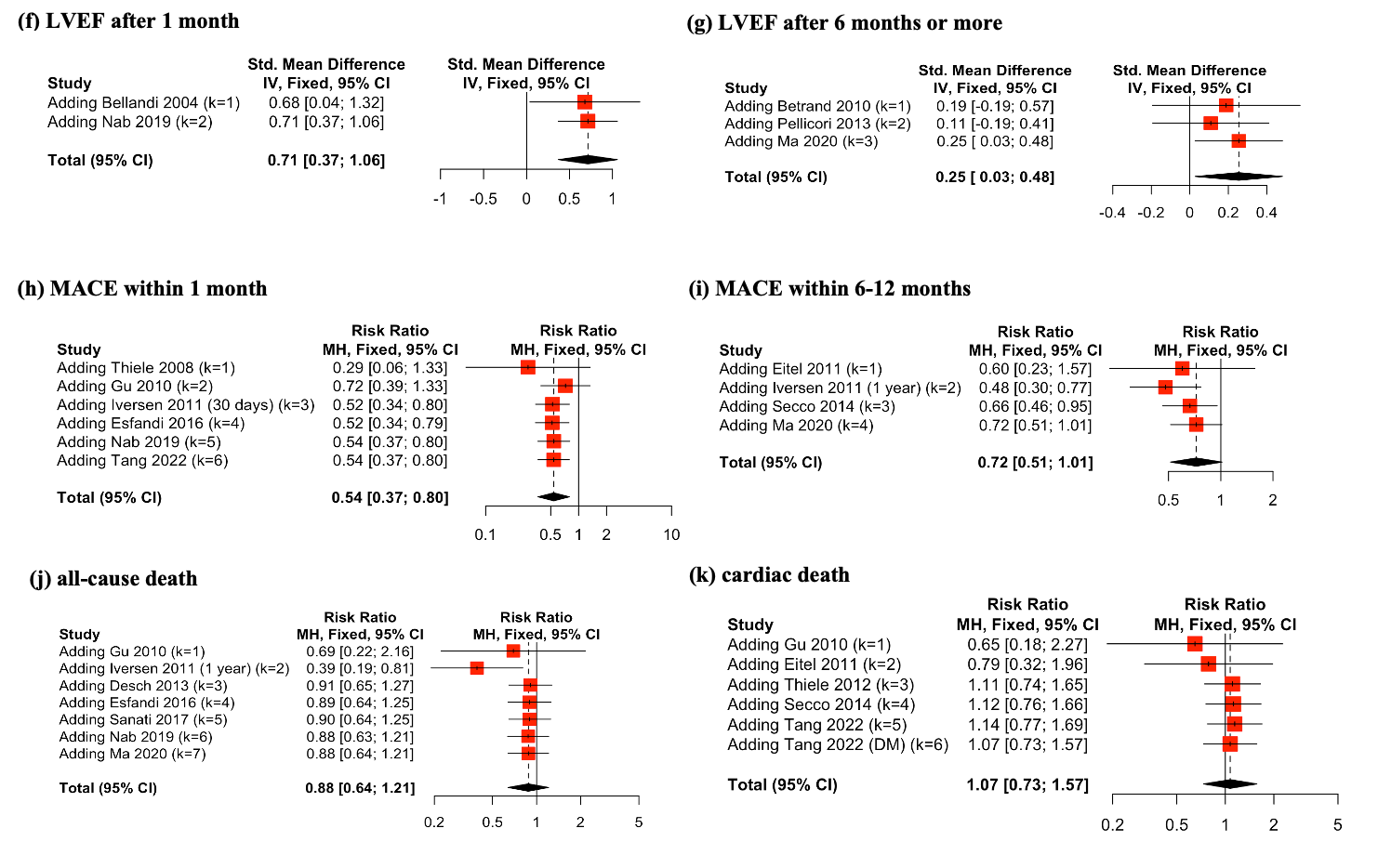


**
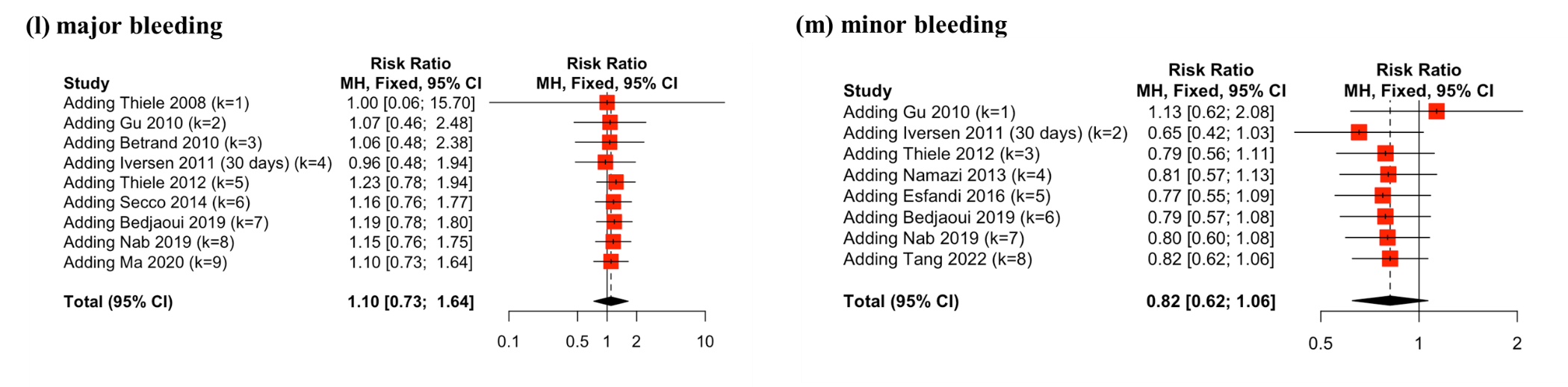
**

Supplementary Figure 8 Sensitivity analysis by publication year
